# Pharmacological Inhibition of Core Regulatory Circuitry Liquid–liquid Phase Separation Suppresses Metastasis and Chemoresistance in Osteosarcoma

**DOI:** 10.1002/advs.202101895

**Published:** 2021-08-25

**Authors:** Bing Lu, Changye Zou, Meiling Yang, Yangyang He, Jincan He, Chuanxia Zhang, Siyun Chen, Jiaming Yu, Kilia Yun Liu, Qi Cao, Wei Zhao

**Affiliations:** ^1^ Key Laboratory of Stem Cells and Tissue Engineering (Sun Yat‐Sen University) Ministry of Education Guangzhou 510080 China; ^2^ Guangdong Provincial People's Hospital Guangdong Academy of Medical Sciences Guangzhou 510080 China; ^3^ Musculoskeletal Oncology Center The First Affiliated Hospital of Sun Yat‐Sen University Guangzhou 510080 China; ^4^ Department of Urology Northwestern University Feinberg School of Medicine Chicago IL 60611 USA; ^5^ Robert H. Lurie Comprehensive Cancer Center Northwestern University Feinberg School of Medicine Chicago IL 60611 USA

**Keywords:** core regulatory circuitry, HOXB8, liquid–liquid phase separation, osteosarcoma, super‐enhancer

## Abstract

Liquid–liquid phase‐separated (LLPS) transcriptional factor assemblies at super‐enhancers (SEs) provide a conceptual framework for underlying transcriptional control in mammal cells. However, the mechanistic understanding of LLPS in aberrant transcription driven by dysregulation of SEs in human malignancies is still elusive. By integrating SE profiling and core regulatory circuitry (CRC) calling algorithm, the CRC of metastatic and chemo‐resistant osteosarcoma is delineated. CRC components, HOXB8 and FOSL1, produce dense and dynamic phase‐separated droplets in vitro and liquid‐like puncta in cell nuclei. Disruption of CRC phase separation decreases the chromatin accessibility in SE regions and inhibits the release of RNA polymerase II from the promoter of SE‐driven genes. Importantly, absence of CRC key component causes a reduction in osteosarcoma tumor growth and metastasis. Moreover, it is shown that CRC condensates can be specifically attenuated by the H3K27 demethylase inhibitor, GSK‐J4. Pharmacological inhibition of the CRC phase separation results in metastasis suppression and re‐sensitivity to chemotherapy drugs in patient‐derived xenograft model. Taken together, this study reveals a previously unknown mechanism that CRC factors formed LLPS condensates, and provides a phase separation‐based pharmacological strategy to target undruggable CRC components for the treatment of metastatic and chemo‐resistant osteosarcoma.

## Graphical abstract

Core regulatory circuitry (CRC) factors forms liquid–liquid phase‐separated condensates at super‐enhancers to regulate chromatin accessibility and oncogenic transcription. Pharmacological inhibition of the CRC phase separation by GSK‐J4 suppresses metastasis and chemoresistance in osteosarcoma patient‐derived xenograft model.

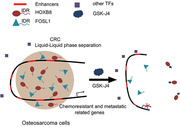

## 1 Introduction

Genetic alterations in cancer cells invariably cause dysregulation in transcriptional programs leading to cancer cells dependency on certain master regulators of oncogene expression, also known as transcriptional addiction.^[^ ^1^ ^]^ This transcriptional addiction is regulated by master transcription factors (TFs) of the core regulatory circuitry (CRC) that are activated and maintained by the large clusters of enhancers, called super‐enhancers (SEs).^[^ ^2^ ^]^ In‐depth analyses of the CRC elucidate putative gene regulatory mechanisms of the cell‐type‐specific gene expression.^[^ ^3^ ^]^ Despite the recent progress of defining SEs in tumors, the CRC in the metastatic and chemoresistant tumors remains poorly understood.

The assembly of dynamic membrane‐free chambers by phase separation is essential for the control of many biochemical processes, including gene transcription.^[^ ^4^ ^]^ It is hypothesized that formation of phase separation more likely occurred at SEs than at typical enchancers (TEs). The transcriptional coactivators BRD4 and MED1 which co‐localized with SEs are components of liquid–liquid phase‐separated (LLPS) condensates.^[^ ^5^ ^]^ The SE‐bound TFs in LLPS condensates typically contain large intrinsically disordered regions (IDRs), which can multivalently but weakly interact with each other. These condensates guarantee the highly concentrated transcriptional machinery at SE regions and play prominent roles in 3D genome organization to robustly activate the expression of cell identity genes.^[^ ^6^ ^]^ An important question related to this is whether CRC components produce LLPS condensates to control the transcriptional addiction‐associated SEs.

Osteosarcoma is the most common primary malignancy of the bone affecting children and adolescents, particularly. Until now, osteosarcoma patients are still receiving treatment that was developed in the 1970s, and the outcomes remain unimproved throughout the years. Although many efforts have been made aiming the oncogenic signaling of osteosarcoma, clinical studies on the targeted agents and immunotherapy yielded disappointing outcomes due to high genetic and pathological heterogeneity of osteosarcoma.^[^ ^7^, ^8^ ^]^ Moreover, metastasis and chemoresistance remain the most important fatal complication of osteosarcoma.^[^ ^9^ ^]^ Nearly all patients succumbed to osteosarcoma develop lung metastases with marked chemoresistance.^[^ ^10^ ^]^ Hence, understanding the mechanisms involving in osteosarcoma metastasis and chemoresistance is urged to develop new strategies and innovative therapies against this disease. As SEs are particularly sensitive to perturbation, agents targeting SEs could be a promising therapeutic options for osteosarcoma. Several studies have shown that inhibitors of chromatin modifiers (e.g., BRD4 inhibitor JQ1) and components of the transcriptional machinery (e.g., CDK7 inhibitor THZ1) diminish the proliferation of patient‐derived cancer cells and increase survival in mouse models through SE‐driven gene inhibition.^[^ ^11^, ^12^ ^]^ The SEs are occupied by high densities of TFs and known to form condensates. We hypothesize that if these SEs can be used as scaffolds, CRC, and transcription machinery proteins will be concentrated at SEs by phase separation to promote metastasis or chemoresistance related gene activation.

**Scheme 1:**
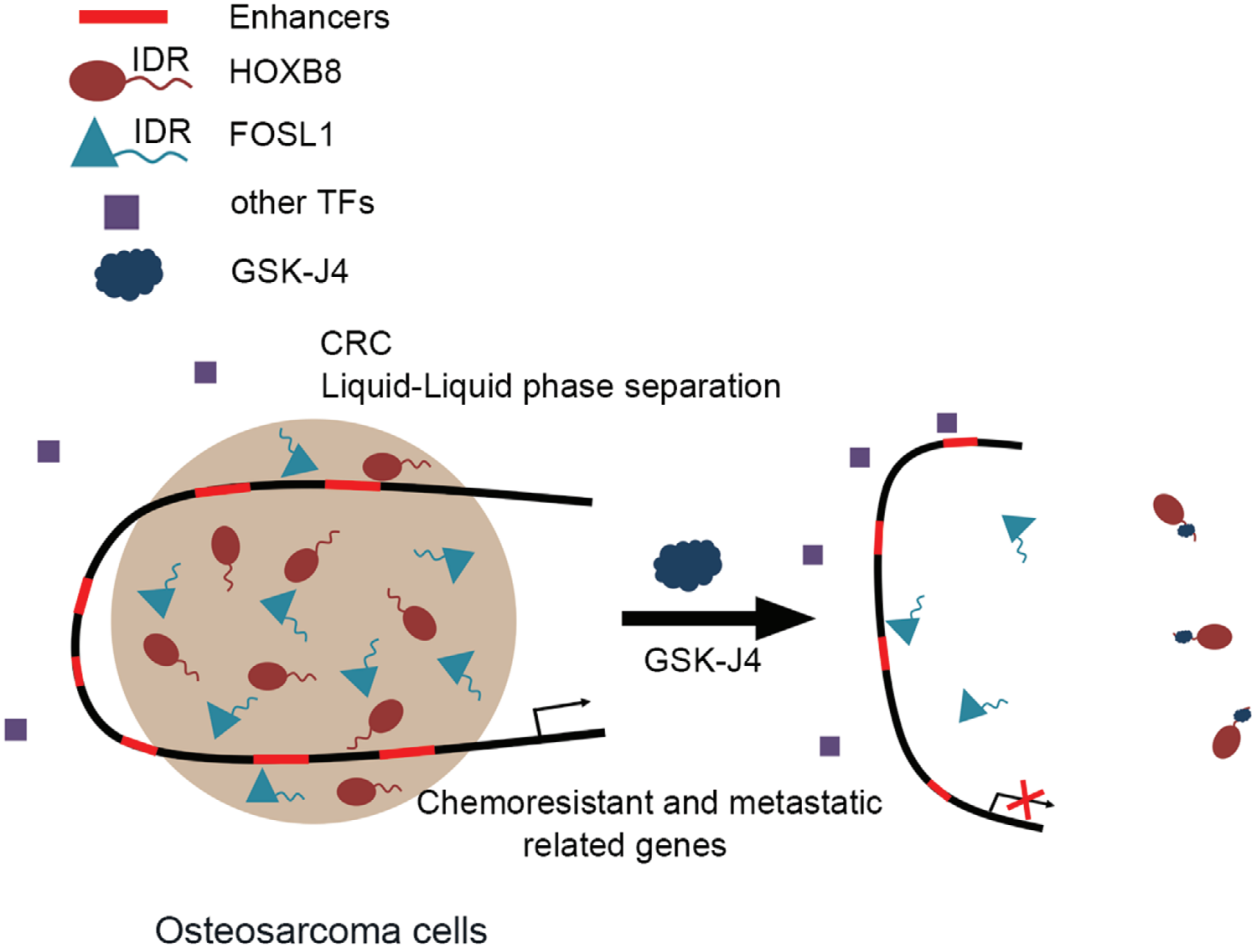
Proposed model of inhibition of CRC condensates mediated oncogenic transcription by GSK‐J4 in chemoresistant and metastatic osteosarcoma.

To this end, we utilized an integrative and large‐scale approach to address how CRC formed condensate to regulate oncogenic transcription in metastatic and chemoresistant osteosarcoma. Furthermore, the insights described in this study can be translated into a better therapeutic strategy using GSK‐J4 by dampening CRC condensate‐mediated transcriptional programs in osteosarcoma with drug‐resistant and metastatic malignancy (**Scheme** **1**).

## 2 Results

### 2.1 SE Landscape Defines Metastasis‐ and Chemoresistance‐Specific CRC

**Figure 1:**
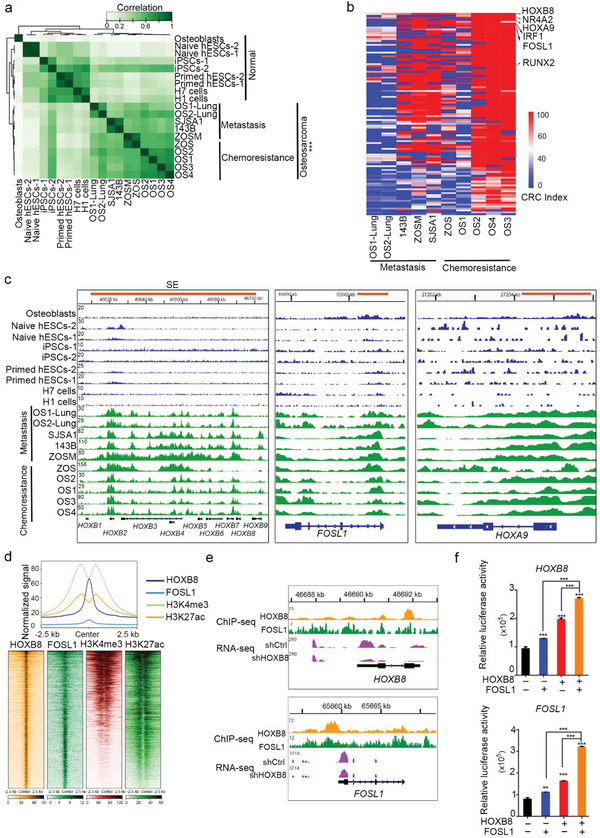
SE landscape defines metastasis‐ and chemoresistance‐specific CRC. a) Unsupervised hierarchical clustering of SE loci detected in osteosarcoma samples (*n* = 10) compared to the normal osteoblasts sample, iPSC samples (*n* = 2), and ESC samples (*n* = 6). b) SE‐driven TFs predicted in a CRC in osteosarcoma. TFs with high IN and OUT degree index are predicated in CRC of osteosarcoma. c) Gene tracks of H3K27ac ChIP‐seq occupancy at CRC related TFs in different types of osteosarcoma samples and normal samples. d) ChIP‐seq data showed co‐occupancy for both HOXB8 and FOSL1 across the genome. Regions co‐bound by HOXB8 and FOSL1 were associated with H3K27ac and H3K4me3 marks in 143B cells. e) Genome browser tracks HOXB8 and FOSL1 binding at HOXB8 and FOSL1 gene loci in 143B cells. f) 293T cells harboring HOXB8 (−873 to +495 bp) or FOSL1 promoter (−970 to +480 bp) ‐fused‐pGL3‐luciferase plasmids were co‐transfected with indicated overexpression plasmid(s). Luciferase activities were tested in these cells on day 2 post‐transfection. ^*^, *p* < 0.05; ^**^, *p* < 0.01; ^***^, *p* < 0.001 is based on the Student's *t*‐test. All results are from more than three independent experiments. Values are mean ± SD.

To pinpoint CRC regulated by SEs in osteosarcoma progression and metastasis, we characterized regions of active chromatin in primary tumors and lung metastases from four chemoresistant patients (Table S1, Supporting Information) using acetylation of histone H3 at lysine 27 (H3K27ac) chromatin immunoprecipitation and sequencing (ChIP‐seq). We also performed ChIP‐seq for H3K27ac in a pair of well‐characterized chemoresistant (ZOS) and metastatic (ZOSM) human osteosarcoma primary cell lines and two metastatic osteosarcoma cell lines (143B and SJSA1). Additionally, we analyzed published ChIP‐seq datasets for H3K27ac in human non‐metastatic and metastatic osteosarcoma cell lines,^[^ ^13^ ^]^ embryonic stem cells (ESCs),^[^ ^14^, ^15^, ^16^ ^]^ induced pluripotent stem cells (iPSCs), and osteoblasts served as normal controls.^[^ ^17^, ^18^ ^]^ We ranked all the putative SEs by increasing the H3K27ac signal and defined SEs as those that showed high H3K27ac enrichment in osteosarcoma (Figure S1a,b, Supporting Information). We identified 3061 and 5405 SEs in the chemoresistant osteosarcoma tumors and metastases, respectively (Figure S1c, Supporting Information). Following KEGG analyses, these SE‐associated genes (identified in both chemotherapy‐resistant and metastatic osteosarcoma tumors) were found to be enriched in RAP1, PI3K/AKT, MAPK, and HIPPO signaling pathways (Figure S1d, Supporting Information). The unsupervised hierarchical clustering analysis based on the acquisition and loss of SE loci, clearly distinguished osteosarcoma from normal cell controls, marked by acquisition and loss of hundreds of SE loci (**Figure** **1**a). SEs were heterogeneous among the osteosarcoma tumor samples, with 63 and 24 overlapping SEs in chemoresistant tumors and metastatic tumors, respectively (Figure S1e, Supporting Information).

The master TFs, which are responsible for transcriptional addiction tend to co‐occupy most SEs together with other master TFs, and typically regulate their own genes through an autoregulatory loop that forms the core transcriptional regulatory circuitry. Therefore, we proposed a definition of osteosarcoma CRC TFs in which the self‐regulated TFs are SE‐driven in osteosarcoma, and the TFs themselves bind to the SEs of one another. The CRC calling algorithms^[^ ^19^, ^20^ ^]^ of osteosarcoma predicted HOXB8 as a top candidate of CRC master TF with the highest IN and OUT degree index in chemoresistant and metastatic osteosarcoma (Figure 1b; Figure S1f, Supporting Information). CRC candidates, HOXB8, FOSL1, and HOXA9, were commonly and specifically associated with SEs in all chemoresistant and metastatic tumor samples (Figure 1c).

Pearson correlation matrix analysis identified HOXB cluster genes, HOXA9, and FOSL1, in the HOXB8 module (Figure S2a, Supporting Information). HOXB8 and FOSL1 ChIP‐seq data (Figure S2b, Supporting Information)further demonstrated that there was a significant co‐occupancy for both HOXB8 and FOSL1 across the genome which were associated with active histone markers, H3K27ac and trimethylation of lysine 4 on histone H3 (H3K4me3) (Figure 1d; Figure S2c, Supporting Information). Notably, 56% of HOXB8/FOSL1 co‐occupancy loci were located at SE‐driven gene regions (Figure S2d, Supporting Information). Furthermore, ChIP‐seq and luciferase reporter assays showed that HOXB8 and FOSL1 regulated their own gene transcription (Figure 1e,f), suggesting that HOXB8 and FOSL1 are self‐regulated and form an interconnected autoregulatory loop (Figure S2e, Supporting Information).

### 2.2 CRC Factors Form Phase‐Separated Droplets In Vitro and Exhibit Liquid‐Like Properties in Cells

**Figure 2:**
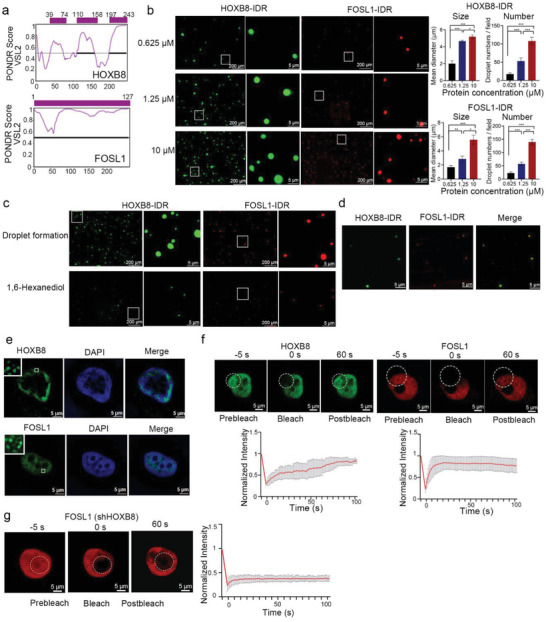
CRC factors form phase‐separated droplets in vitro and exhibit liquid‐like properties in cells. a) Graphs plotting intrinsic disorder (PONDR VSL2) for HOXB8 and FOSL1. PONDR VSL2 score (*y*‐axis) and amino acid position (*x*‐axis) are shown. Purple bar designates the IDR under investigation. b) Representative images of droplet formation at different protein concentrations. HOXB8‐IDR, FOSL1‐IDR were added to droplet formation buffer to final concentrations indicated. Scale bar = 5 µm. c) Representative images of droplet formation at 10 µm protein HOXB8‐IDR, FOSL1‐IDR before and after addition of vehicle or 1,6‐hexanediol to a final concentration of 3%. Scale bar = 5 µm. d) HOXB8‐IDR droplets incorporate FOSL1‐IDR protein in vitro. The indicated mEGFP or mCherry fusion proteins were mixed at 1.25 µm. Scale bar = 5 µm. e) Immunofluorescence (IF) imaging of HOXB8 and FOSL1 in 143B cells. Fluorescence signal and DAPI stain is shown. Scale bar = 5 µm. f) Representative images of FRAP experiment of mEGFP‐HOXB8 and mCherry‐FOSL1 engineered 143B cells. Quantification of FRAP data for mEGFP‐HOXB8 puncta and mCherry‐FOSL1 puncta. Bleaching event occurs at *t* = 0 s. For both bleached area and unbleached control, background‐subtracted fluorescence intensities are plotted relative to a pre‐bleach time point (*t* = −5 s). Scale bar = 5 µm. g) Representative images of FRAP experiment of mCherry‐FOSL1 in HOXB8 knock down cells. Quantification of FRAP data for mCherry‐FOSL1 puncta. Bleaching event occurs at *t* = 0 s. For both bleached area and unbleached control, background‐subtracted fluorescence intensities are plotted relative to a pre‐bleach time point (*t* = −5 s). Scale bar = 5 µm. ^*^, *p* < 0.05; ^**^, *p* < 0.01; ^***^, *p* < 0.001 is based on the Student's *t*‐test. All results are from more than three independent experiments. Values are mean ± SD.

CRC factors HOXB8 and FOSL1 contain large IDRs (**Figure** **2**a) which are known to contribute to the formation of phase separation condensates. So we purified recombinant mEGFP‐HOXB8‐IDR and mCherry‐FOSL1‐IDR fusion proteins and performed the droplet formation assay with varying concentrations (from 0.625 to 10 µm). mEGFP‐HOXB8‐IDR and mCherry‐FOSL1‐IDR formed spherical droplets in a concentration dependent manner (Figure 2b). We observed that LLPS of HOXB8‐IDR and FOSL1‐IDR was dampened by treatment of 1,6‐hexanediol or elevated salt concentration (Figure 2c; Figure S3a, Supporting Information), suggesting these droplets are reversible phase‐separated condensates. In addition, purified full‐length HOXB8 and FOSL1 formed micron‐sized droplets which can also be inhibited by 1,6‐hexanediol (Figure S3b,c, Supporting Information). Mixing mEGFP‐HOXB8‐IDR and mCherry‐FOSL1‐IDR proteins resulted in droplets that contain both proteins (Figure 2d). Direct interactions between HOXB8 and the FOSL1 may contribute to the phase separation (Figure S3d,e, Supporting Information).

Fixed cell immunofluorescence (IF) revealed that both HOXB8 and FOSL1 formed puncta in 143B cells (Figure 2e; Figure S3f, Supporting Information). The dynamic recombination and fast exchange kinetics of liquid‐like condensates formed by HOXB8 and FOSL1 were determined by measuring the fluorescence recovery rate (FRAP) after photobleaching. After photobleaching, mEGFP‐HOXB8 or mCherry‐FOSL1 puncta recovered fluorescence on a time‐scale of seconds (Figure 2f). HOXB8 is required for the LLPS formation of CRC condensates, because liquid‐like properties of FOSL1 disappeared in HOXB8 knockdown (KD) cells (Figure 2g).

### 2.3 Disruption of CRC Phase Separation Decreases Chromatin Accessibility at SE Loci and Impairs RNA Polymerase II Elongation on SE‐Driven Genes

**Figure 3:**
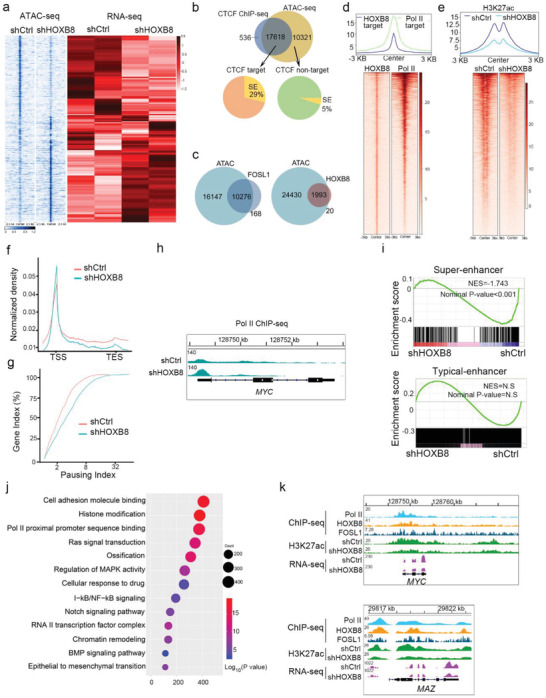
Disruption of CRC phase separation decreases chromatin accessibility and impairs RNA Polymerase II (Pol II) elongation at SE‐driven gene loci. a) Expression of HOXB8 target genes relative to appearing and disappearing ATAC‐seq peaks. b) Pie diagram showing the percentage of SE‐driven genes in CTCF target genes and non‐target genes with changed accessibility in 143B cells. c) Venn diagram overlap of HOXB8 or FOSL1 ChIP binding genes with accessible genes in 143B cells. d) ChIP‐seq data showed co‐occupancy for both HOXB8 and Pol II across the genome in 143B cells. e) Heatmap showing the signal intensity of H3K27ac of HOXB8 targets in control (shCtrl) and shHOXB8. Regions bound by HOXB8 were associated with decreased H3K27ac marks in shHOXB8 compared with shCtrl in 143B cells. f) Metagene profile of Pol II for HOXB8 targets in 143B cells treated with shCtrl and shHOXB8. g) Line graph of the pausing index (PI) for HOXB8 targets upon HOXB8 silencing versus shCtrl. h) Genome browser tracks Pol II binding at *MYC* gene loci in shCtrl and shHOXB8 RNA‐seq data. i) GSEA analysis plot of the SE‐associated and TE‐associated gene sets compiled from 143B and SJSA1 cells derived from shHOXB8 versus those derived from shCtrl. j) GO terms of genes commonly associated with decreasing H3K27ac signal of HOXB8 targeted genes. k) Genome browser tracks of Pol II, HOXB8, and FOSL1 ChIP‐seq data, shCtrl and shHOXB8 H3K27ac and RNA‐seq data in SE‐driven gene (*MYC* and *MAZ*) locus. ^*^, *p* < 0.05; ^**^, *p* < 0.01; ^***^, *p* < 0.001 is based on the Student's *t*‐test. All results are from more than three independent experiments.

Recent studies have suggested that liquid–liquid phase separation contributed to higher‐order chromatin structure.^[^ ^5^ ^]^ Chromatin Interaction Analysis with Paired‐End‐Tag sequencing (ChIA‐PET) data suggests that SE constituents occupied by polymerase II (Pol II), HOXB8, and FOSL1 are close to each other in the 3D structure of chromatin, exampled by *MAZ* and *MYC* (Figure S4a, Supporting Information). The CRC occupied transcription active regions of open chromatin were delineated by assay for transposase‐accessible chromatin using sequencing (ATAC‐seq). Disruption of CRC phase separation via depletion of HOXB8 resulted in significant changes of chromatin architecture with disappearing peaks associated with downregulated gene expression (**Figure** **3**a). Following GO analyses, these downregulated genes were found to be enriched in cell–cell adhesion and cell growth signaling pathways (Figure S4b, Supporting Information). Motif scanning using i‐cisTarget revealed that the changed ATAC peaks were highly enriched for FOSL1 and CTCF motifs (Figure S4c, Supporting Information). Using CTCF ChIP‐seq data, we found that the 29% changed (ATAC‐seq peaks increased or decreased) CTCF accessible sites are SEs (Figure 3b). To determine whether chromatin accessible sites are enriched for CRC TFs occupancy, we overlapped the ATAC‐seq peaks with HOXB8 or FOSL1 binding peaks. The majority of HOXB8 or FOSL1 binding occurred at genes with accessible chromatin regions (Figure 3c). These characteristics may engender SE sites as particularly vulnerable to disruption of CRC phase separation.

HOXB8 was identified as a factor that co‐occupied with Pol II across the genome (Figure 3d). We also detected a significant reduction in H3K27ac modification on the loci of HOXB8 targets in HOXB8‐depleted 143B cells (Figure 3e). Although we observed an increase of Pol II density at the promoters of HOXB8 target SE‐driven genes, there was a widespread loss of Pol II presence at these gene bodies (Figure 3f). Next, we calculated the Pol II pausing index (PI, also known as a traveling ratio) with the Pol II ChIP‐seq data. The results revealed a significant upregulation of PI, indicative of impaired elongation, at SE‐driven genes in shHOXB8 143B cells compared to the shCtrl cells (Figure 3g). For many SE‐driven genes, exemplified by *MYC*, reduction in HOXB8 expression leads to decrease of Pol II within gene bodies and H3K27ac modification at enhancer regions (Figure 3h). These results indicate that CRC LLPS condensates promote the elongation of transcription of SE‐driven genes.

To further elucidate the mechanisms underlying CRC LLPS dependency in SE driven gene transcription, we conducted RNA‐seq after silencing HOXB8 in different osteosarcoma cell lines. RNA‐seq analysis identified 653 deregulated genes upon HOXB8 depletion in both osteosarcoma cell lines (Figure S4d,e, Supporting Information). The mRNA levels of SE‐driven genes, in particular, HOXB8 binding SE‐driven genes, reduced markedly upon HOXB8 silencing (Figure 3i). CRC LLPS inhibition affected the H3K27ac of HOXB8 targeted genes associated to GO terms linked to cell adhesion molecule binding and cellular response to the drug (Figure 3j), such as SE driven genes *MYC* and *MAZ* (Figure 3k).

### 2.4 CRC Phase Separation is Essential in Osteosarcoma Growth and Metastasis

**Figure 4:**
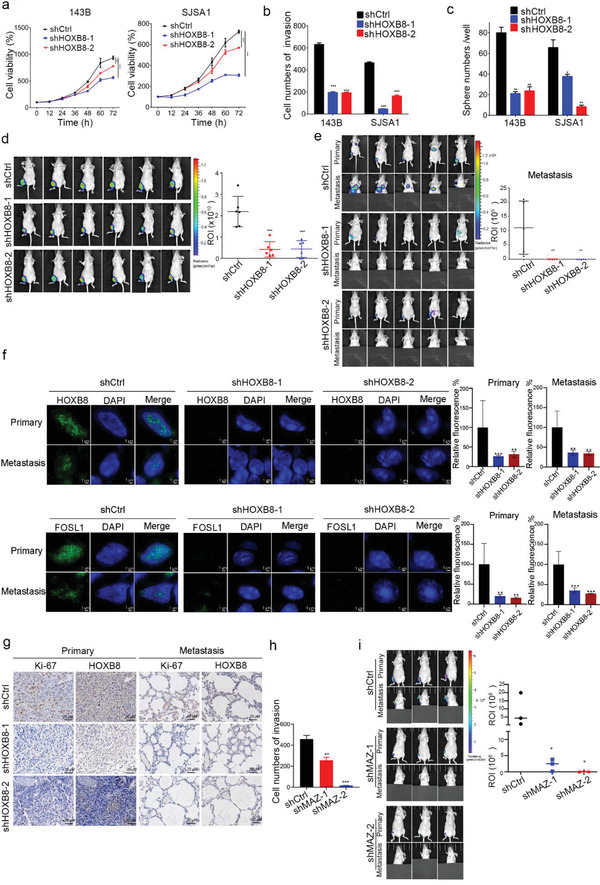
CRC is essential in osteosarcoma growth and metastasis. a) Relative growth curves of 143B and SJSA1 cells stably transduced with non‐targeting scrambled control shRNA (shCtrl) or two HOXB8 shRNAs (shHOXB8‐1 and shHOXB8‐2), respectively. Values are normalized to control (day 0). b) Invasion assay was conducted in shHOXB8 versus shCtrl by using 24‐well Transwell chambers. Cell invasion was assessed by counting the number of migrated cells after 24 h. c) Sphere forming assay was performed on 143B and SJSA1 cells with or without shHOXB8 transduction. The spheroids were counted on day 7. d) 1.5 ×  10^6^ luciferase transduced 143B cells with KD of HOXB8 (shHOXB8‐1, shHOXB8‐2) or shCtrl were used to establish an orthotopic model of osteosarcoma. Luminescence was observed using an in vivo imaging system (IVIS) after cell inoculation at week 4 (*n* = 6 per group). e) 1 ×  10^6^ 143B cells with shHOXB8 or shCtrl, respectively, were used to establish an orthotopic metastatic model of osteosarcoma. Luminescence was observed using an IVIS after cell inoculation at week 6 (*n* = 5 per group). The representative images of metastasis are shown with the primary tumors covered. f) High‐resolution images of HOXB8 and FOSL1 in the mouse xenograft primary tumors and lung metastases by immunofluorescence staining on frozen tissues. g) Ki67 and HOXB8 expression in orthotopic injection model tumor tissues (from H) by immunohistochemistry (IHC) staining assay. Scale bar = 20 µm. h) Invasion assay was conducted in sh*MAZ* versus shCtrl by using 24‐well Transwell chambers. Cell invasion was assessed by counting the number of migrated cells after 24 h. i) 1.5  × 10^6^ luciferase transduced 143B cells with KD of *MAZ* or shCtrl were used to establish an orthotopic model of osteosarcoma. Luminescence was observed using IVIS after cell inoculation at week 4 (*n* = 3 per group). The representative images of metastasis are shown with the primary tumors covered. ^*^, *p* < 0.05; ^**^, *p* < 0.01; ^***^, *p* < 0.001 is based on the Student's *t*‐test. All results are from more than three independent experiments. Values are mean ± SD.

To further document the consequence of disruption of CRC phase separation on osteosarcoma, we stably suppressed HOXB8 expression using two distinct shRNAs (shHOXB8‐1 and shHOXB8‐2; Figure S5a, Supporting Information). Importantly, CRC LLPS inhibition via HOXB8 silencing (Figure S5b,c, Supporting Information) impaired osteosarcoma cell proliferation, invasion, and sphere formation in 143B and SJSA1 cells (**Figure** **4**a–c). To investigate the consequences of CRC LLPS inhibition in vivo, 143B cells stably expressing control shRNA or shHOXB8 were orthotopically injected into the central cavity of the bone of immunocompromised mice. Cells expressing shHOXB8 showed significant reduction in tumor growth versus controls (Figure 4d). Moreover, overexpression of HOXB8 promoted osteosarcoma cell invasion (Figure S5d, Supporting Information). We performed a spontaneous metastasis experiment using an orthotopic injection model and found that HOXB8 knockdown significantly blocked metastasis (Figure 4e), consistent with reduced propensity of HOXB8 and FOSL1 to undergo LLPS in primary and metastasis sites (Figure 4f). The ratios of Ki67‐positive cells and the numbers of metastatic tumor cells were lowered in HOXB8 KD cell‐derived primary and metastatic tumors (Figure 4g).

As a newly identified SE‐driven gene in metastatic osteosarcoma, the biological roles of MAZ in osteosarcoma remain unclear. To determine whether MAZ is a crucial downstream effector in CRC dependent transcription facilitating the metastasis, we silenced *MAZ* in 143B cells (Figure S5e, Supporting Information). Our results showed that depletion of *MAZ* reduced osteosarcoma invasive activity (Figure 4h) and inhibited the metastasis in the orthotopic tumor xenograft mouse model (Figure 4i). Taken together, we concluded that CRC LLPS inhibition significantly reduced metastasis at least partly via the MAZ in osteosarcoma.

### 2.5 H3K27me3 Demethylase Inhibitor, GSK‐J4, Disrupts the CRC Condensates

**Figure 5:**
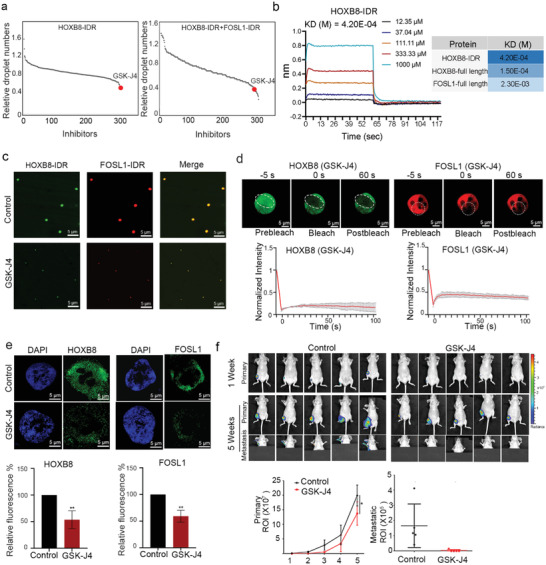
H3K27me3 demethylase inhibitor, GSK‐J4, disrupts the CRC condensates. a) The relative number of HOXB8‐IDR and FOSL1‐IDR droplets compared with the dimethyl sulfoxide (DMSO) control group after 24 h of treatment with the compound library, the screening concentration was 10 µm. All assays were performed in the presence of 62.5 mm NaCl and 10% PEG‐8000 was used as a crowding agent. b) Bio‐layer interferometry (BLI) assays were performed with purified HOXB8‐IDR protein (or HOXB8/ FOSL1 full length protein) and GSK‐J4. Biotin‐labeled proteins were immobilized on the streptavidin biosensors and dipped into wells containing increasing concentrations of GSK‐J4. c) Representative images of droplet formation at 0.625 µm protein HOXB8‐IDR, FOSL1‐IDR with the incubation of DMSO control and 10 µm GSK‐J4. Scale bar = 5 µm. d) Representative images of FRAP experiment of mEGFP‐HOXB8 or mCherry‐FOSL1 in GSK‐J4 treated cells (5 µm, 48 h). Quantification of FRAP data for mCherry‐FOSL1 puncta. Bleaching event occurs at *t* = 0 s. For both bleached area and unbleached control, background‐subtracted fluorescence intensities are plotted relative to a pre‐bleach time point (*t* = −5 s), Scale bar = 5 µm. e) Representative images of HOXB8 and FOSL1 condensates upon 10 µm GSK‐J4 treatment in 143B cells. Scale bar = 5 µm. f) The 143B cell‐derived orthotopic model of osteosarcoma mice was administrated intraperitoneally with 100 mg kg^−1^ GSK‐J4 10 days in a row (once a day). Luminescence was observed using an IVIS at weeks 1 and 5 (*n* = 5 per group). The representative images of metastasis are shown with the primary tumors covered. Primary tumor growth and metastasis of 143B‐derived orthotopic tumors were calculated based on the region of interest (ROI) value. ^*^, *p* < 0.05; ^**^, *p* < 0.01; ^***^, *p* < 0.001 is based on the Student's *t*‐test. All results are from more than three independent experiments. Values are mean ± SD.

The emergence of LLPS provides a new strategy to target undruggable CRC proteins, such as HOXB8 and FOSL1. After three‐round screening of 303 chemicals, we found that the phase‐separated HOXB8‐IDR and FOSL1‐IDR condensates could be specifically and selectively disrupted by the treatment of a trimethylation of H3K27 (H3K27me3) demethylase inhibitor, GSK‐J4 (**Figure** **5**a). Bio‐layer interferometry (BLI) assays confirmed that GSK‐J4 directly binds to HOXB8‐IDR (Figure 5b).

GSK‐J4 also inhibited the CRC condensates produced by the co‐aggregation of HOXB8‐IDR and FOSL1‐IDR (Figure 5c). Photobleaching of GSK‐J4 treated condensate could not result in a recovery of HOXB8 or FOSL1 fluorescence (Figure 5d), which is consistent with the inhibited liquid‐like behavior of HOXB8 KD. Moreover, in GSK‐J4 treated cells, the punctas of HOXB8 and FOSL1 were markedly decreased in the nuclei (Figure 5e).

Importantly, GSK‐J4 inhibited the proliferation of various osteosarcoma cell lines at clinically achievable concentrations and showed limited efficacy against normal mesenchymal stem cell and osteoblast cell proliferation at the same dosage (Figure S6a,b, Supporting Information). The number of metastatic nodules to the front limb and surface of the lungs was completely inhibited in the GSK‐J4‐treated mice (Figure 5f; Figure S6c, Supporting Information). GSK‐J4 treatment also led to less bone damage (Figure S6d, Supporting Information). IHC staining of primary xenografts and metastatic lungs revealed that the GSK‐J4 treatment groups exhibited barely detectable immunoreactivity for HOXB8 and Ki67 (Figure S6e, Supporting Information). Importantly, GSK‐J4 treatment did not show any signs of toxicity to major organs or affect animal body weight status (Figure S6f,g, Supporting Information).

We next investigated how GSK‐J4 treatment affects genome‐wide SE‐driven gene expression using RNA‐seq analysis (Figure S7a, Supporting Information). Gene set enrichment analysis (GSEA) revealed that the SE‐driven genes were enriched in transcripts downregulated after GSK‐J4 treatment (Figure S7b, Supporting Information). *MYC* and *MAZ* were among the most profoundly affected SE‐driven genes (Figure S7c, Supporting Information). Moreover, since HOXB8 form an auto‐regulatory loop by binding to its own SEs, GSK‐J4 treatment also inhibits the expression of HOXB8 (Figure S7d,e, Supporting Information).

### 2.6 Pharmacological Modulation of CRC Phase Separation Confers Additional Advantages to Circumvent Chemo‐Resistance

**Figure 6:**
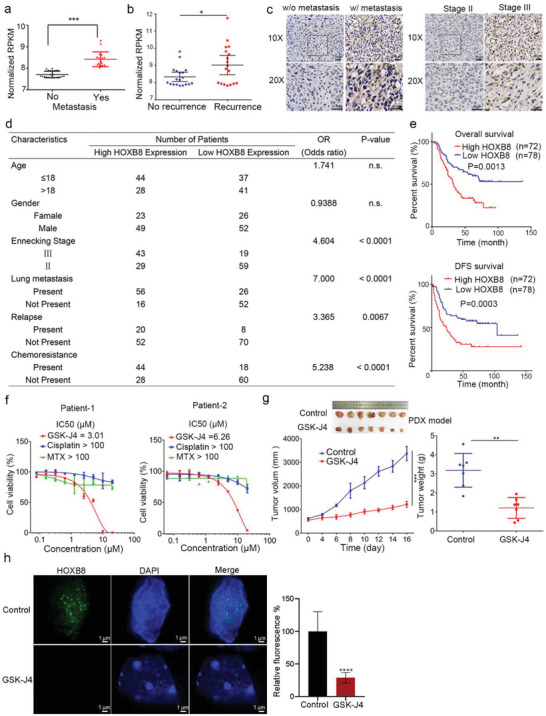
Pharmacological modulation of CRC phase separation confers additional advantages to circumvent chemo‐resistance. a,b) HOXB8 expression in nonmetastatic samples (*n* = 24) versus metastatic samples (*n* = 18) ((a) data from GSE33383) and non‐recurrent samples (*n* = 19) versus recurrent samples (*n* = 18) ((b) data from GSE39055). c) Representative immunohistochemistry (IHC) images of HOXB8 in tumors without (w/o) metastasis versus tumor with (w/) metastatic (upper), and Ennecking stage II versus stage III (lower) of osteosarcoma. Scale bar, 20 µm. d) Statistical table of 150 osteosarcoma patients by HOXB8 staining intensity. Significance was determined by a chi‐squared test. e) Kaplan–Meier overall survival curve of 150 osteosarcoma patients by HOXB8 IHC score. Patients with high HOXB8 expression levels (*n* = 72) had poor prognosis, whereas patients with low HOXB8 IHC expression levels (*n* = 78) had favorable prognosis. f) Cell viability assays were performed with patient‐derived primary cells treated with GSK‐J4, MTX, or cisplatin for 48 h. g) Representative images of tumor tissue (day 16) in vehicle control and 50 mg kg^−1^ GSK‐J4 treatment groups of PDX model (*n* = 7). The PDX tumor growth curve and weight are shown. h) Representative high‐resolution images of HOXB8 condensates in PDX tumors treated with (*n* = 4) or without (*n* = 3) GSK‐J4. Scale bar = 1 µm. ^*^, *p* < 0.05; ^**^, *p* < 0.01; ^***^, *p* < 0.001 is based on the Student's *t*‐test. All results are from more than three independent experiments. Values are mean ± SD.

Next, the phase separation of HOXB8 was validated in osteosarcoma clinical specimens (Figure S8a, Supporting Information). To explore whether the osteosarcoma metastasis correlated with CRC components expression, we analyzed HOXB8 expression data from gene expression omnibus databases. Significantly higher levels of HOXB8 were found in metastatic osteosarcoma tumors compared with non‐metastatic tumors (**Figure** **6**a). Intriguingly, increased expression of HOXB8 was also observed in recurrent tumors compared with primary tumors (Figure 6b). To determine the correlations between the HOXB8 expression status and chemo‐resistance, we stained for HOXB8 in osteosarcoma clinical specimens (*n* = 150; Table S1, Supporting Information) that comprised of specimens representing primary progression to chemoresistant disease and stratified them based on grade and histology. The expression of HOXB8 was upregulated in metastatic and poorly differentiated tumors (Figure 6c). Tumor stage (*p* < 0.0001), lung metastasis (*p* < 0.0001), tumor relapse (*p* = 0.0067), and chemoresistance (*p* < 0.001) are positively correlated with HOXB8 staining (Figure 6d; Figure S8b, Supporting Information). Moreover, osteosarcoma patients with a high level of HOXB8 expression exhibited a lower overall survival and disease‐free survival than the patients with a low level of HOXB8 expression (Figure 6e). These results demonstrate that HOXB8 expression level have important prognostic significance for the osteosarcoma patients undergoing chemotherapy.

To further explore the therapeutic potential of GSK‐J4 for osteosarcoma with methotrexate (MTX) and cisplatin resistance, we generated two chemoresistant human primary cell lines (Patient‐1 and Patient‐2) derived from osteosarcoma patients (Figure S8c, Supporting Information) those were resistant to MTX and cisplatin therapy (Figure 6f). In a short‐term proliferation assay, Patient‐1 and Patient‐2 cells proliferated in the presence of MTX and cisplatin, respectively, whereas Patient‐1 and Patient‐2 cells were sensitive to GSK‐J4 (Figure 6f). The benefit of GSK‐J4 was validated in MTX‐ and cisplatin‐resistant osteosarcoma patients (same patient as Patient‐1 cells)‐derived xenograft (PDX) models (Figure 6g). Moreover, GSK‐J4 attenuated number of HOXB8 condensates in the nuclei of PDX tumor cells (Figure 6h). Taken together, GSK‐J4 suppressed chemoresistant osteosarcoma by disturbing CRC LLPS.

## 3 Discussion

Tumor SE‐driven master TFs in CRC are enriched at oncogenic driver genes that play prominent roles in malignancies, including metastasis and drug resistance.^[^ ^21^ ^]^ Therefore, CRC has recently been highlighted as attractive targets for epigenetic therapy of cancer. How these master TFs of CRC aggregate and orchestrate at SEs to generate downstream outcomes is still an unanswered question. Here, we demonstrated that formation of phase‐separated condensates is an important mechanism that enables CRC‐mediated transcription addiction in metastasis and chemoresistance. In particular, we present a small molecule compound, GSK‐J4, which modulates phase separation of CRC factors, for therapeutic intervention of chemoresistant and metastatic osteosarcoma.

Our study clarifies the role of HOXB cluster genes, in particular HOXB8, as the master TFs of CRC in metastasis and chemoresistance. Previous studies showed that HOXB8 functions as a key TF involved in limb development and bone marrow‐derived microglia differentiation. Our study reveals that the osteosarcoma malignancy exhibits a dependence on CRC TFs (e.g., HOXB cluster genes and FOSL1)‐mediated oncogenic transcriptional programs. This type of transcriptional dependency appears to operate in many certain types of cancers. For example, recent studies have shown that HOXB cluster genes can be classified as SEs in acute myeloid leukemia, and HOXB cluster gene expression is dependent on mutant NPM1 and its aberrant cytoplasmic localization.^[^ ^22^ ^]^ Another study showed that a retinoid‐dependent cis‐regulatory element, distal element RARE,^[^ ^23^ ^]^ determined HOXB cluster gene expression, and leukemic state. It is an open question why HOXB, the important TFs during limb development, promote osteosarocma malignancy if expressed in maligant cells. Our study suggests that phase separation may play an important role in the modulation of transcription specificity of HOXB8 by driving assembly of HOXB8 with oncogenic TFs in the “oncogenic” condensate and preventing binding of HOXB8 with original development related TFs. The CRC condensates where HOXB8 is located provides a new approach for the formation of transcription addiction related to metastasis and chemoresistance.

The majority of Pol II‐transcribed genes are regulated at a checkpoint called promoter‐proximal pausing. In this study, we found that the inhibition of CRC could block the release from pausing during transcription, leading to transcriptional elongation suppression in metastatic osteosarcoma cells. Previous publications also demonstrated MYC hyperactivation induces transcriptional amplification by increasing mRNA synthesis. Notably, MYC functions to amplify the entire gene expression program, which is a critical step for generating transcriptional addiction in cancers. Intriguingly, the newly identified SE‐driven gene MAZ may assist MYC in metastasis and functions as another important effector of CRC in transcriptional addiction. The downstream targets of MAZ in osteosarcoma need to be further identified.

We identified that H3K27me3 demethylase inhibitor, GSK‐J4, suppressed the phase separation of CRC factors. An attractive hypothesis is that GSK‐J4 reduces HOXB8 expression by inhibiting JMJD3/UTX which are putative nuclear epigenetic factors required for H3K27me3 demethylation. But the cell‐free assays showing HOXB8‐mEGFP forming little phase separated droplets after GSK‐J4 treatment makes this scenario unlikely. Therefore, we speculate that GSK‐J4 binds IDR of HOXB8 could somehow affect HOXB8 protein folding. Moreover, treatment of GSK‐J4 may further suppress the expression of SE‐driven oncogenic genes in osteosarcoma. One prime example of SE inhibitors is the small molecule inhibition of histone deacetylases (e.g., Panobinostat) in diffuse intrinsic pontine glioma, for which clinical trials are ongoing. Our results demonstrate that, in addition to histone acetylation, the CRC phase separation may present additional avenues for therapeutic intervention in malignancies with aberrant changes in the oncogenic SEs. Notably, although GSK‐J4 targets HOXB8 to disrupt CRC phase separation, we cannot exclude the contribution of other targets to the therapeutic activity of GSK‐J4 in osteosarcoma.^[^ ^24^ ^]^

In summary, we identify the chemoresistant‐ and metastatic‐specific CRC formed phase‐separated condensates at SE loci in osteosarcoma. The disruption of CRC phase separation suppressed aberrant oncogenic transcriptional programs via modulating chromatin accessibility. Moreover, a small molecule compound, GSK‐J4, can be used as a potential treatment for metastatic and chemoresistant osteosarcoma, which will greatly benefit osteosarcoma patients.

## 4 Experimental Section

### Osteosarcoma Specimen and Bone Tissue Collection

Both osteosarcoma and normal bone tissue surgical specimens were collected in the Department of Bone Tumor, the First Affiliated Hospital, Sun Yat‐sen University in accordance with institution‐approved protocols. Written informed consent was obtained from each study participant after a thorough explanation of the procedure and its risk according to the Declaration of Helsinki. All specimens were examined by a pathologist to verify tumor types and grades.

### Cell Cultures

The human osteosarcoma cell lines of 143B, SJSA1, U2, MNNG, ZOS, and ZOSM were cultured in DMEM medium (Corning, USA) with 10% fetal bovine serum (FBS; BI, USA) at 37 °C in a humidified 5% CO_2_ atmosphere.

For the culture of primary osteosarcoma cells and osteoblasts, surgically removed osteosarcoma specimens and normal bone tissue were washed with and minced in sterile phosphate‐buffered saline (PBS). The single‐cell suspension was obtained by pressing the minced tissues through 70‐µm cell strainers (Falcon, USA). Dissociated cells were cultured in DMEM medium supplemented with 15% FBS (Gibco, USA) at 37 °C in a humidified 5% CO_2_ atmosphere.

### Animals

Female Balb/c nude mice (18 ± 2 g, 4–6 weeks) were obtained from the Model Animal Research Center of Nanjing University (China). All animal experiments were carried out according to the guidance of the ethics committee of Sun Yat‐Sen University.

### Subcutaneous Xenograft Model

Five‐week‐old female Balb/c athymic nude mice were housed in individually ventilated micro‐isolator cages. Each mouse was injected subcutaneously in the right flank with 10^6^ osteosarcoma cells in 100 µL PBS. Thereafter, tumor size was periodically measured with calipers every 2 days by measuring the length and width. Tumor volumes were calculated according to the following formula: volume (mm^3^) = (length × width × width) / 2. After the mice were sacrificed, the tumor xenografts were removed, fixed in formalin, and stored at 4 °C. Animal experiments were approved by the Animal Care and Use Committee of Sun Yat‐sen University.

### Orthotopic Xenograft Model

Single‐cell suspension (10 µL) containing 1–1.5 × 10^6^ osteosarcoma cells was injected into the right tibial medulla of chloral hydrate‐anesthetized 5‐week‐old nude mice. Five weeks later, using an in vivo imaging system (IVIS; Xenogen), mice were imaged under isoflurane anesthesia to analyze osteosarcoma tumor growth in vivo.

### Patient‐Derived Xenograft Model

Tumor specimens were obtained from osteosarcoma patients with their informed consent. Tumor fragments were removed during surgery and saved on ice for less than 45 min. Briefly, fresh tumor fragments were grafted subcutaneously into the subcutaneous tissue upper back of NCG (NOD‐Prkdc^em26^Il2rg^em26^/Nju) mice under anesthesia with 10% chloral hydrate. Xenografts appeared at the graft site 1 to 3 months after grafting. They were subsequently transplanted from mouse to mouse. Animals were excluded if no tumors were present, and animals were randomized to control and treatment groups so that each group had equivalent distribution of initial tumor sizes.

### Constructs of shRNA and Viral Packages

The shRNA sequences against target genes were designed and cloned into the pLKO‐puro lentiviral vector. To generate lentiviral particles, the constructed shRNA expression plasmid was co‐transfected with packaging plasmids pVSVg and psPAX2 into human embryonic kidney 293T cells using lipofectamine 2000 (Invitrogen, USA). 143B and SJSA1 osteosarcoma cells were infected with the obtained lentiviruses to knockdown target genes. The shRNA‐targeting sequences are listed in Table S2, Supporting Information.

### ChIP

ChIPs were performed using 2 × 10^6^ to 10 × 10^6^ cross‐linked cells, and sequencing libraries were prepared as previously described. The following antibodies were used for ChIP: rabbit anti‐H3K4me3 (Abcam), rabbit anti‐H3K27ac (Abcam), rabbit anti‐H3K27me3 (CST), and rabbit anti‐HA (CST). ChIP‐seq libraries were sequenced on the HiSeq 2000 platform at the Novogene LLC. Analysis was performed as previously described.

### RNA Sequencing

All treatment conditions were collected in biological duplicate. Cells were lysed in TRIzol reagent and frozen at −80 °C. Total RNA was extracted using RNeasy Mini kit (QIAGEN, 74106) following the user's manual and quantified using Nanodrop 2000. The integrity of the RNA was analyzed using the Agilent Bioanalyzer 2100. Paired‐end reads were generated by Illumina Hi‐Seq 2500 platform and mapped to the human genome. An R package, DESeq, was applied for transcription quantification and differential expression analysis using a cutoff of *p* < 0.05. Changes in splicing isoforms were analyzed by replicate multivariate analysis of transcript splicing, a Bayesian statistical framework.

### ChIP‐Seq Data Pre‐Processing, Enhancer, and SE Analysis

Trim_galore and Fastqc were used for trimming adapters and filtering raw sequencing reads. Sequencing reads were aligned to the UCSC hg19 human genome reference using bowtie2. All unmapped reads, non‐uniquely mapped reads, and PCR duplicates were removed. ChIP‐seq peaks were then called using MACS2. Correlation of ChIP‐seq samples were calculated by R/Bioconductor package DiffBind. Chip peak motif discovery was performed using findMotifsGenome.pl. Genome coverage files for visualization were generated by igvtools using count command and default options. Chip peak browsing and representative snapshots capturing were performed using the Integrative Genomics Viewer.

### SE Analysis

We identified SEs using the Rank Ordering of Super Enhancers (ROSE) algorithm (https://bitbucket.org/young_computation/rose). The SE was classified as a set of H3K27ac peaks (detected by MACS2) within a 12.5 kb distance, and greater than 2.5 kb distance from the transcriptional start site (TSS).^[^ ^11^ ^]^ Super enhancers were further defined by those demonstrating the greatest levels of H3K27 acetylation as detected by graphing an inflection plot and selecting values for which the slope of a fitted curve exceeded a value of 1. Enhancers below the point on that curve with a slope of 1 were thus defined to be typical‐enhancers.

### Calculating CRC for SE Associated TFs

Osteosarcoma CRC analysis was determined using the COLTRON (https://pypi.python.org/pypi/coltron) and CRCmapper (https://github.com/younglab/CRCmapper) that calculated IN and OUT degree index for SE‐regulated TFs. Briefly, for any given TF, the IN degree index was defined as the number of SE‐driven TFs with a binding at the given TF proximal SE. The OUT degree index was defined as the number of binding sites for SE‐driven TFs. An FDR cutoff of 0.01 was used to identify enriched TF binding sites. The TF in CRC was defined as the number of IN and OUT degree index was greater than 150.

### Pausing Index Calculation

PI is the ratio between the normalized coverage of Pol II in the gene body (from TSS to tanscriptional termination site) and the normalized coverage of Pol II in the promoter of genes (defined as ±500 bp from the TSS).

### Nucleosome Spacing Calculation

Paired‐end ATAC‐seq fragments of 180–247 bp were isolated and the average dyad density at single nucleotide resolution was plotted around FOSL1 motifs. The spacing between mono‐nucleosomes was determined by the maximum labeling density of upstream and downstream nucleotides around the FOSL1 motif.

### Fluorescence Recovery after Photobleaching (FRAP)

A lentiviral overexpression plasmid for HOXB8 was generated by cloning the full‐length ORF of human HOXB8 gene (NM_024016.4) into the FG‐EH‐DEST‐FLAG‐MCS vector, followed by a 6 amino acid GS linker sequence “GSGSGS” and mEGFP. FOSL1 expression plasmid was generated by cloning the full‐length ORF of human FOSL1 gene (NM_005438.5) with 5′HA tag into the FG‐EH‐DEST‐FLAG‐MCS vector followed by a 6 amino acid GS linker mentioned above and mCherry. FRAP was performed on LSM880 Airyscan microscope with 488 nm laser. The bleach spot was centered on a cluster and images were taken with 1 s interval for 100 s to measure the fluorescence recovery in the cluster. The integrated intensity of the cluster was determined as a function of time, background intensity was subtracted from a neighboring region of equal size, was corrected for overall photo‐bleaching based on a reference region within the same cell, and was normalized to pre‐bleach intensity.

### Quantification and Statistical Analysis

All the in vitro experiments were tested at least in triplicate. All analyses were performed using GraphPad Prism version 6.0 (GraphPad Software, USA). Data were presented as the mean ± SD. Unpaired two‐tailed *t*‐test was performed to compare the statistical significance of all tests. Chi‐squared test, one‐way and multivariate ANOVA were performed to compare clinical data. *p*‐values of less than 0.05 were considered statistically significant and marked as “^*^”; *p*‐values less than 0.01 or 0.001 were marked as “^**^” and “^***^,” respectively. All data needed to evaluate the conclusions in the paper are present in the paper and/or the Supporting Information. Additional data are available from authors upon request.

## Conflict of Interest

The authors declare no conflict of interest.

## Author contributions

B.L. and C.Z. contributed equally to this work. B.L. performed the experiments and wrote the manuscript. C.Z. performed the xenograft experiments and IHC staining assay. M.Y. and Y.H. performed LLPS assays. J.H. conducted bioinformatic analysis of sequencing data. C.X.Z. performed the co‐IP assays. S.C. and J.Y. prepared the plasmids and lentiviral vectors. K.Y.L. and Q.C. interpreted the data and revised the manuscript. W.Z. designed the experiments, interpreted the data, wrote the manuscript, and provided supervision.

## Supporting information

Supporting Information

## Data Availability

The data that support the findings of this study are available from the corresponding author upon reasonable request.
