# Supplementary material for: Pharmacological Inhibition of Core Regulatory Circuitry Liquid–liquid Phase Separation Suppresses Metastasis and Chemoresistance in Osteosarcoma
Source: Adv Sci (Weinh). 2021 Aug 25;8(20):2101895. doi: 10.1002/advs.202101895 (PMC8529446; doi:10.1002/advs.202101895)
Supplement: Supplementary file 1 — Supporting Information [file ADVS-8-2101895-s001.pdf]

Supporting Information

Pharmacological Inhibition of Core Regulatory Circuitry Liquid-liquid Phase Separation Suppresses Metastasis and Chemoresistance in Osteosarcoma

Bing Lu, Changye Zou, Meiling Yang, Yangyang He, Jincan He, Chuanxia Zhang, Siyun Chen, Jiaming Yu, Kilia Yun Liu, Qi Cao, and Wei Zhao

Contents

Supplemental Figures 1-8

Supplemental Tables 1-3

Supplementary Materials and Methods

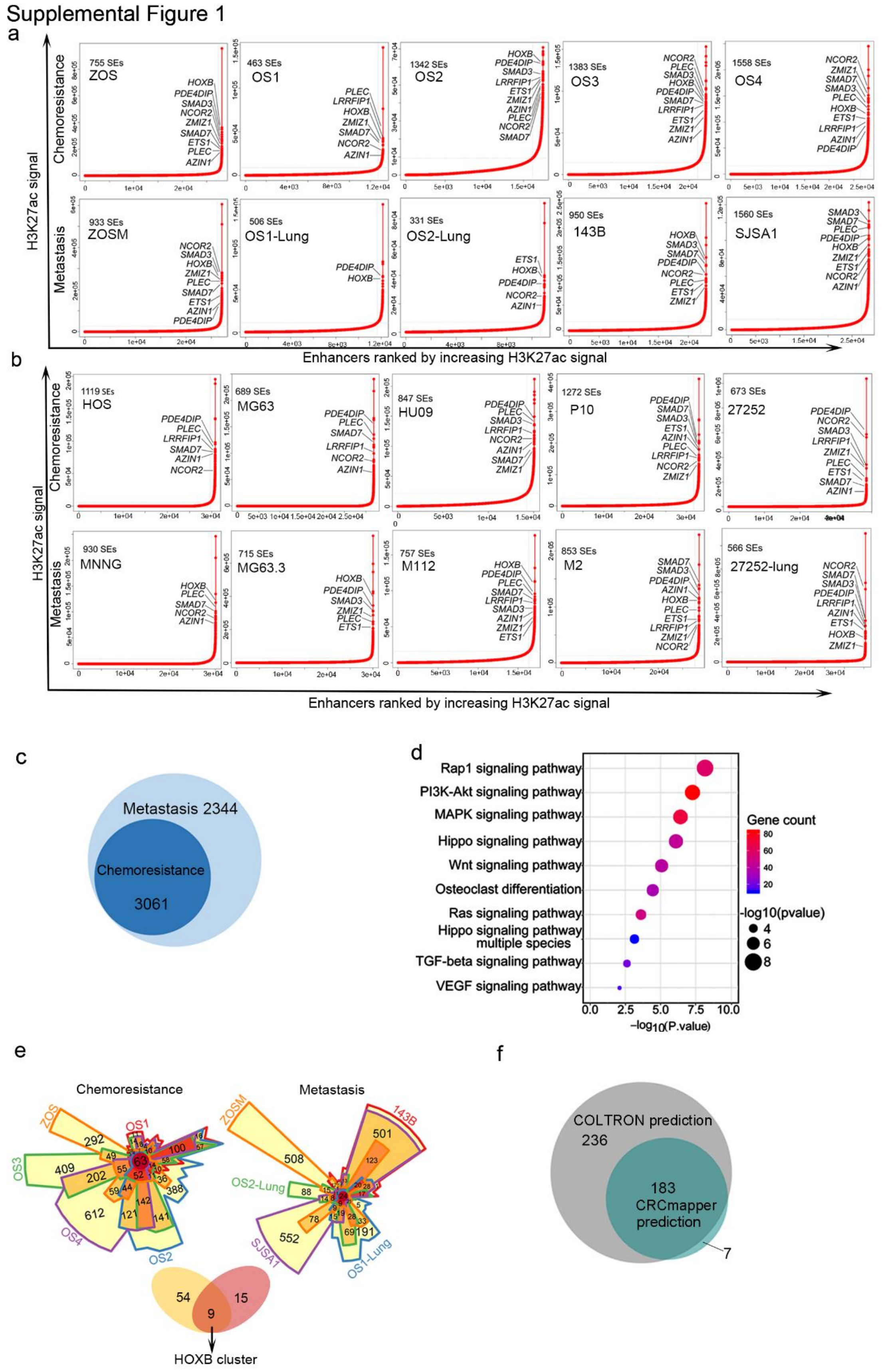

Figure S1. Super-Enhancer (SE) landscape defines metastasis- and chemoresistance-specific core regulatory circuitry (CRC). Related to Figure 1.

a) Enhancers were ranked by increasing H3K27ac signal in four osteosarcoma cell lines (143B, SJSA1, ZOS, and ZOSM), four chemoresistant osteosarcoma specimens (OS1, OS2, OS3, and OS4), and two osteosarcoma lung metastasis specimens (OS1-Lung and OS2-Lung). The number of SEs is shown for each sample. Examples of genes that were commonly associated with SEs in at least four samples are shown.

b) Enhancers were ranked by increasing H3K27ac signal in five primary osteosarcoma samples (HOS, MG63, HU09, P10, 27252) and five metastatic osteosarcoma samples (MNNG, MG63.3, M112, M2, 27252-Lung). The number of SEs is shown for each sample. The data are from GEO database GSE74230.

c) Venn diagrams depict the number of shared SEs between the chemoresistant (*n*=5) and metastatic (*n*=5) osteosarcoma samples.

d) Genes commonly associated with SEs in parental and metastatic cases were subjected to gene ontology analysis and KEGG pathway analysis.

e) Upper, Integrative analysis of chemoresistant and metastatic samples co-regulated by SEs. The number indicates SE-associated genes in osteosarcoma samples. Lower, Venn diagrams depicting the number of shared SE-associated genes between the chemoresistant and metastatic osteosarcoma samples.

f) The number of CRC transcriptional factors (TFs) predicted by COLTRON and CRCmapper calling algorithms.

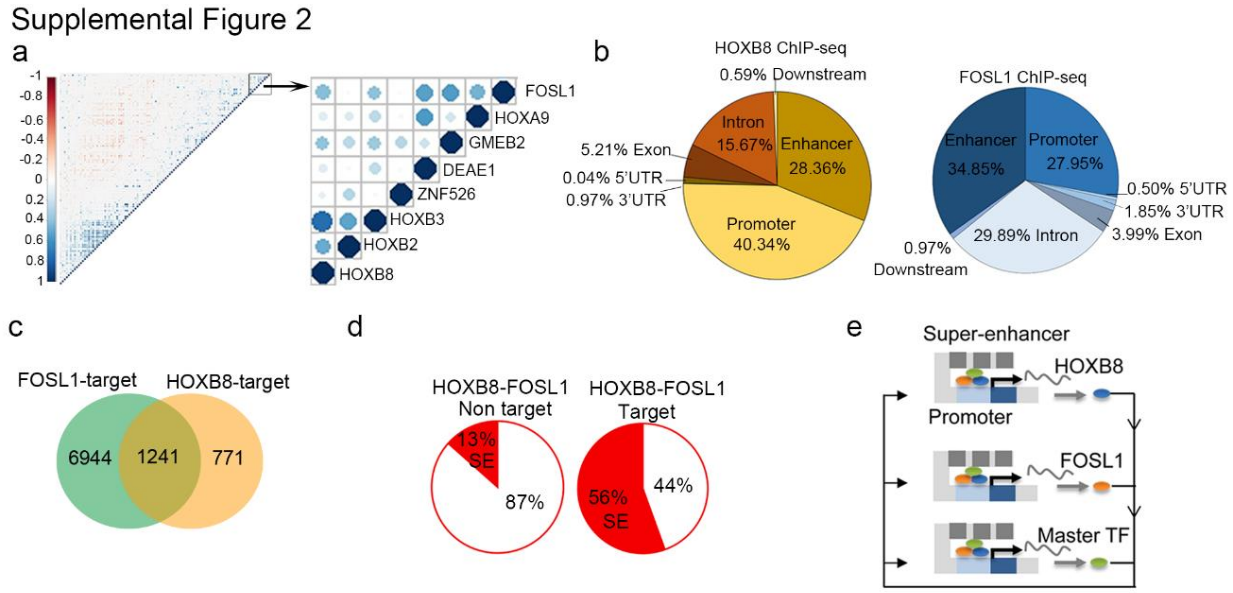

Figure S2. HOXB8 and FOSL1 co-occupy SE-driven genes and form autoregulatory loops. Related to Figure 1.

a) Pearson correlation matrix of the SE-driven TFs in a set of osteosarcoma tumors (data from GSE87624).

b) Genome-wide distribution of HOXB8 and FOSL1 ChIP-seq peaks in 143B cells.

c) Venn diagram showing the overlap of HOXB8 and FOSL1 target genes in 143B cells.

d) Pie diagram showing the percentage of SE-driven genes in HOXB8 and FOSL1 co-target genes and non-target genes in 143B cells.

e) Schematic diagram of autoregulatory loops of CRC in chemotherapy-resistant and metastatic osteosarcoma.

Supplemental Figure 3

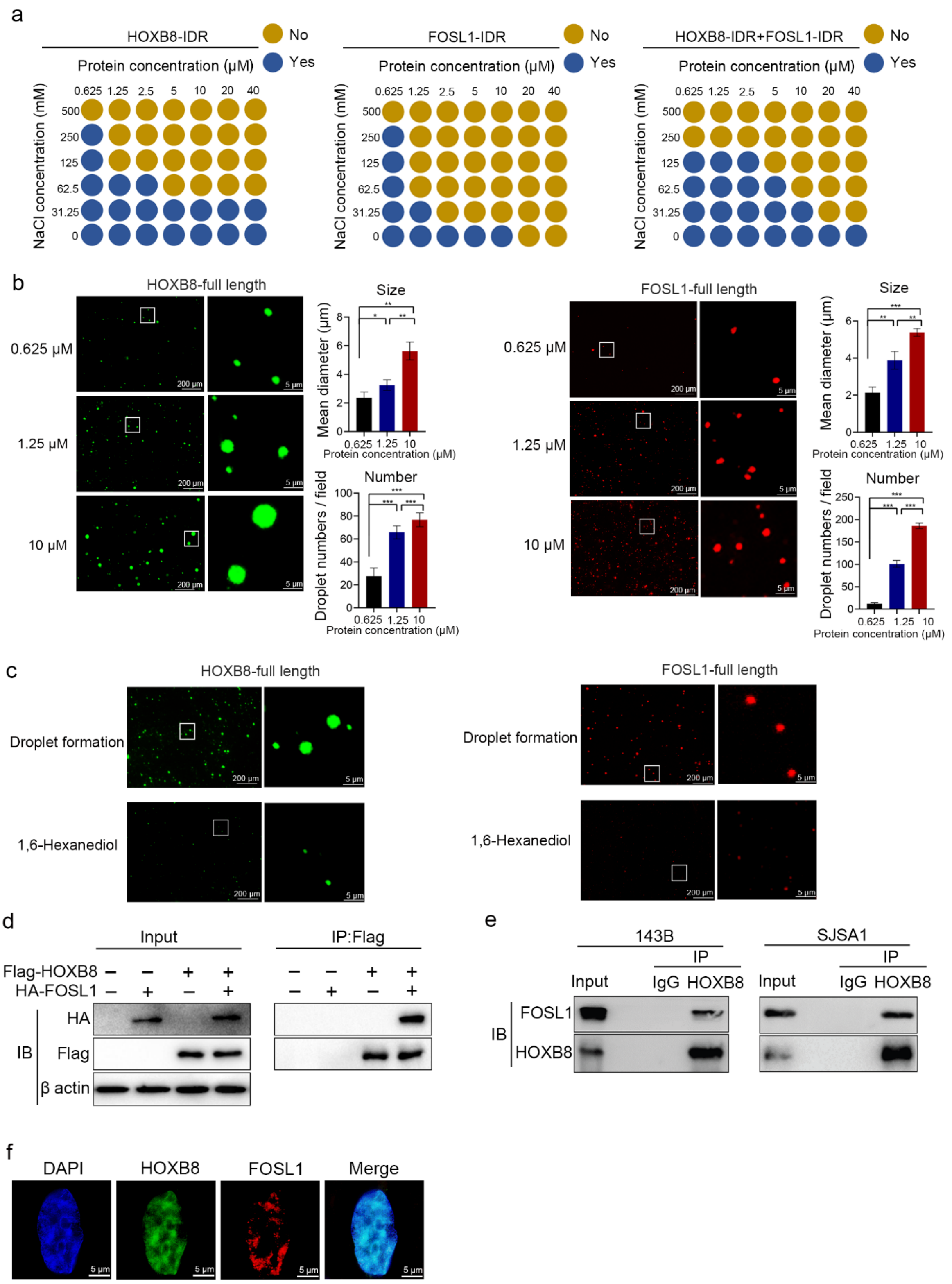

Figure S3. Full-length of CRC factors form liquid droplets . Related to Figure 2.

a) Phase diagrams for HOXB8-IDR , FOSL1-IDR or the mixture of HOXB8-IDR and FOSL1-IDR showing concentrations of salt (ranging from 0-500 mM) and protein (ranging from

0.625-40 μM). Yellow dots, no liquid-liquid phase separation; blue dots, liquid-liquid phase separation.

b) Representative images of HOXB8/FOSL1 full length-formed droplets at different protein concentrations. Scale bar = 5 μm.

c) Representative images of droplets formed by HOXB8- full length or FOSL1- full length protein upon 3% 1,6-hexanediol treatment. Scale bar = 5 μm.

d) 293T cells were transfected with Flag-tagged HOXB8 or/and HA-tagged FOSL1. Cell extracts were immunoprecipitated with anti-Flag beads, followed by immunoblotting with the anti-HA and anti-Flag antibody.

e)143B or SJSA1 cell extracts were immunoprecipitated with anti-HOXB8 antibody and protein A/G beads, followed by immunoblotting with anti-FOSL1 and anti-HOXB8 antibody.

f) Representative images of overexpressed mEGFP-HOXB8 (full length) and mCherry-FOSL1(full length) in 143B cells. Nucleus is shown by DAPI staining. Scale bar = 5 μm.

*, $P < 0.05$; **, $P < 0.01$; ***, $P < 0.001$ is based on the Student's t test. All results are from more than three independent experiments. Values are mean ± SD.

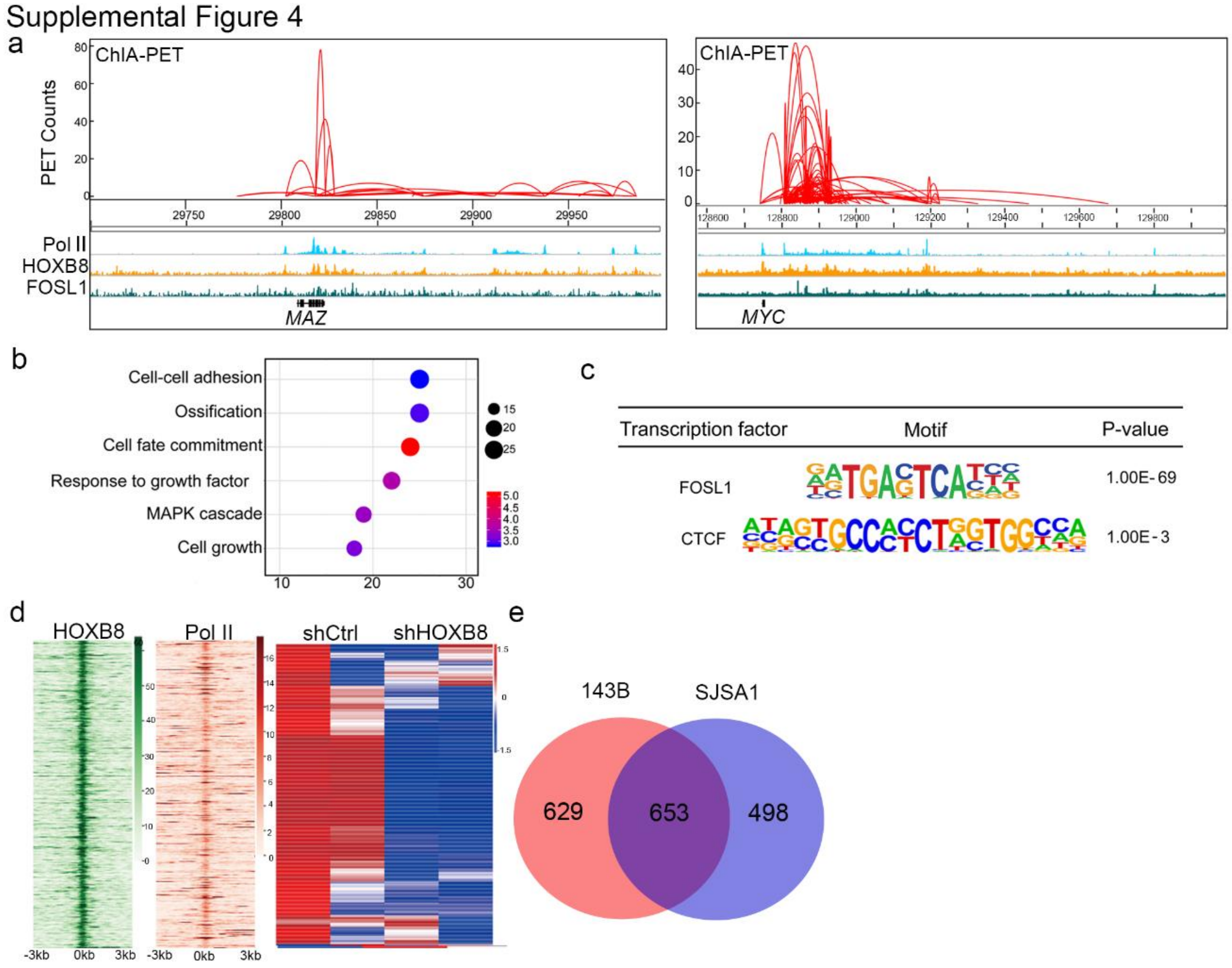

Figure S4. SE-driven gene MAZ is a critical downstream target of CRC in the process of osteosarcoma metastasis. Related to Figure 3.

a) Depiction of *MAZ* and *MYC* locus, associated DNA contacts (red arcs) from ChIA-PET data (data from GEO:GSM970209), Pol II, HOXB8 and FOSL1 ChIP-seq.

b) Gene ontology (GO) analysis of the decreased ATAC-seq peaks associated genes upon knockdown HOXB8.

c) DNA motifs enriched in ATAC-seq peaks derived from knockdown HOXB8 143B cells by HOMER motif analysis.

d) Heatmap showing differential gene expression in shCtrl and shHOXB8 143B cells.

e) Venn diagram showing the overlap of 653 significantly expressed genes between shCtrl and shHOXB8 in both 143B and SJSA1 cells.

Supplemental Figure 5

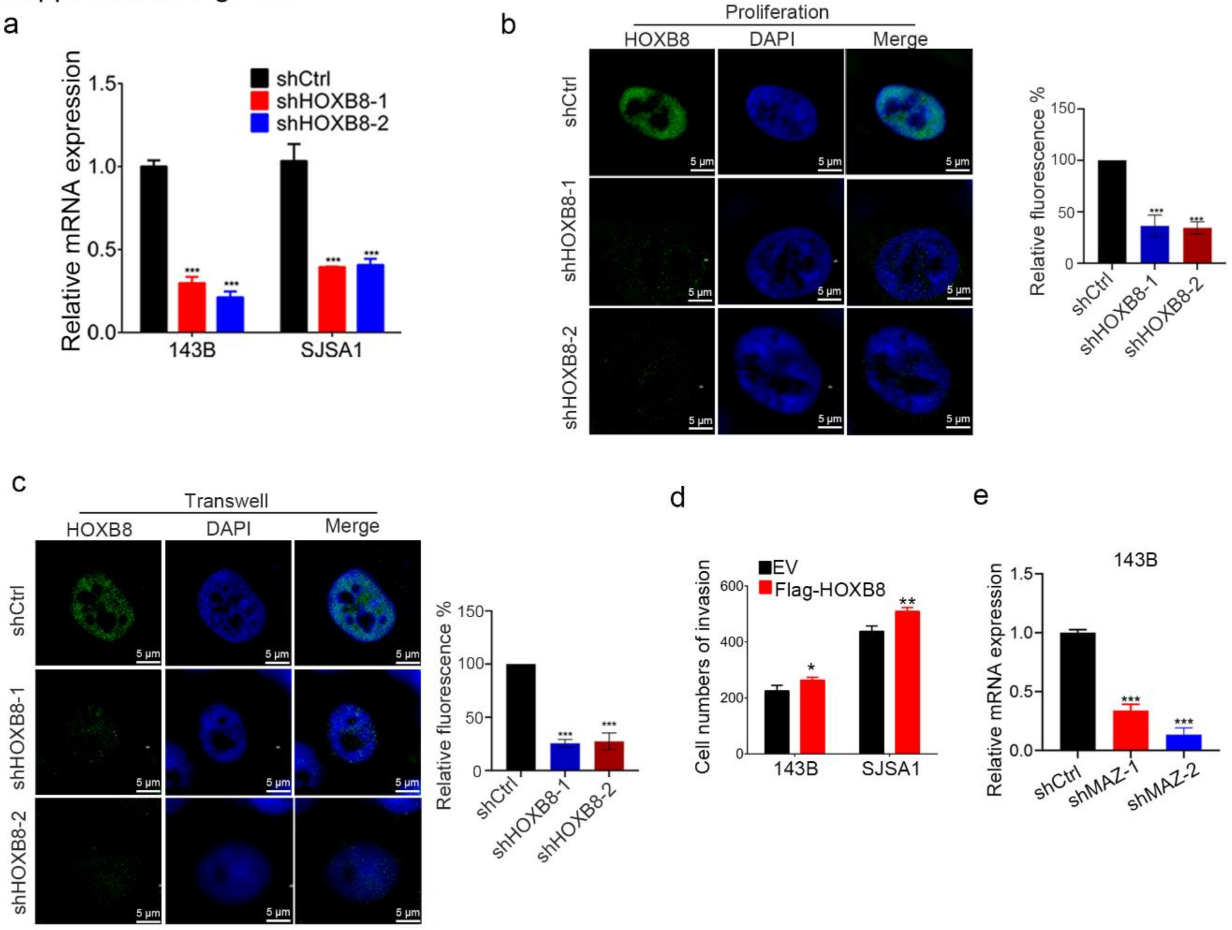

Figure S5. Knockdown (KD) of HOXB8 suppresses osteosarcoma invasion by disrupting the CRC phase separation. Related to Figure 4.

a) qPCR showing KD efficiency of HOXB8 in 143B and SJSA1 cells.

b and c) Representative fluorescence microscopy images of HOXB8 puncta in 143B cells from proliferation assays (b) and transwell assays (c). Scale bar = 5 μm.

d) Invasion assay was conducted in HOXB8 overexpression vs. empty vector by using 24-well transwell chambers. Cell invasion was assessed by counting the number of migrated cells after 24 h.

e) qPCR showing KD efficiency of MAZ in 143B cells.

*, $P < 0.05$; **, $P < 0.01$; ***, $P < 0.001$ is based on the Student's t test. All results are from more than three independent experiments. Values are mean ± SD.

Supplemental Figure 6

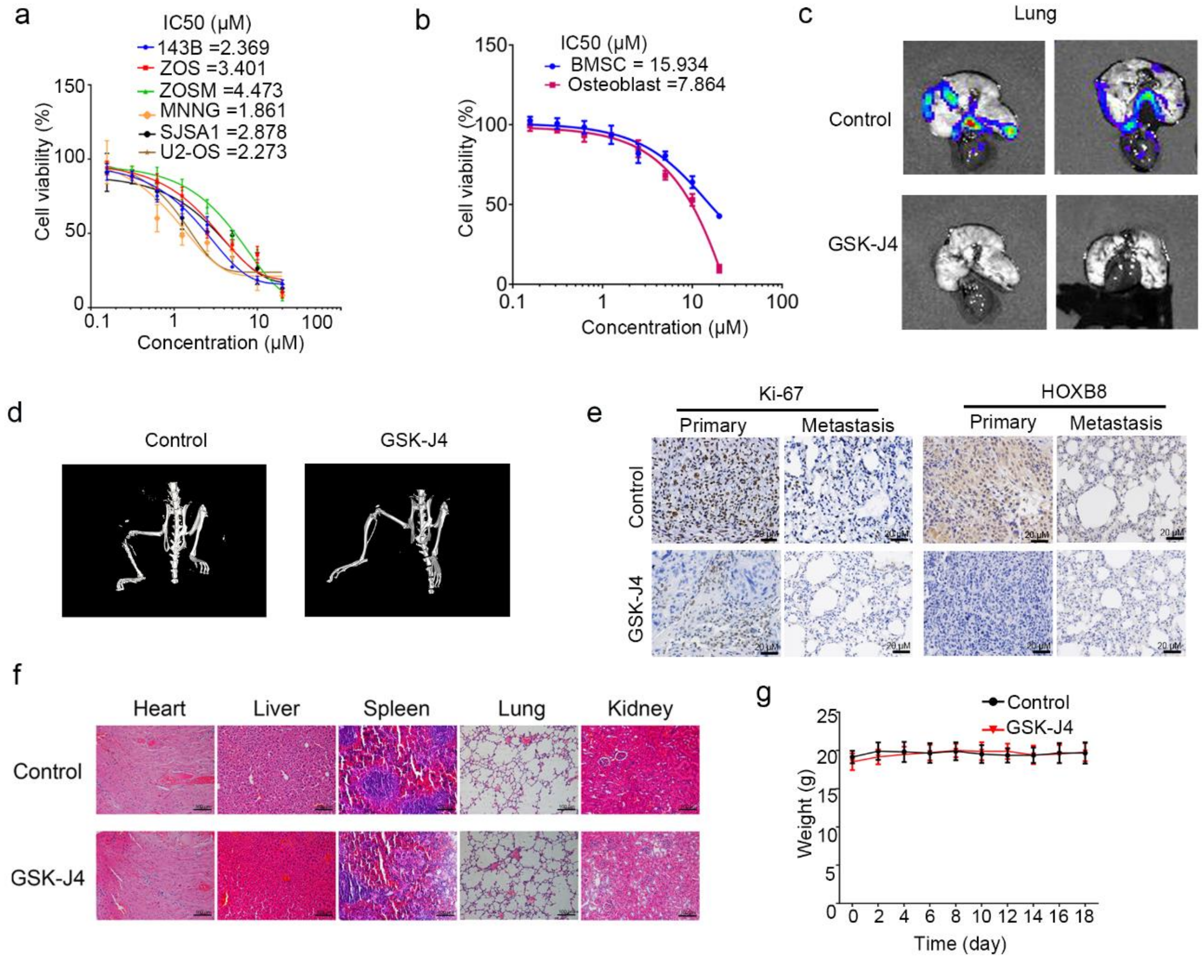

Figure S6. CRC phase separation inhibitor GSK-J4, suppresses growth and metastasis of osteosarcoma cells. Related to Figure 5.

a) Cell viability assay showing osteosarcoma cells (143B, ZOS, ZOSM, MNNG, SJSA1, and U2-OS) cultured for 48 h in the presence of GSK-J4 at the indicated concentration. Cell growth was compared to the DMSO control.

b) Cell viability assay of bone marrow-derived mesenchymal stem/stromal cells (BMSCs) and primary osteoblasts cultured for 48h in the presence of GSK-J4 at the indicated concentration.

c) Representative luminescence pictures for control and GSK-J4-treated lungs from orthotopic osteosarcoma model mice (n=5 per group).

d) Representative computed tomography (CT) picture for control and GSK-J4-treated bones from

orthotopic osteosarcoma model mice (n=5 per group).

e) Representative Ki-67 and HOXB8 staining for control and GSK-J4-treated tumors from orthotopic osteosarcoma model mice (n=5 per group). Scale bar = 20 μm.

f) H&E stained images of heart, liver, spleen, lung, and kidney in 100 mg kg^{-1} GSK-J4 i.p. treated and control mice (n=5 per group). Scale bar = 100 μm.

g) The body weight of nude mice (n=5 per group) with subcutaneous xenograft intraperitoneal injected (i.p.) with or without 100 mg kg^{-1} GSK-J4.

Supplemental Figure 7

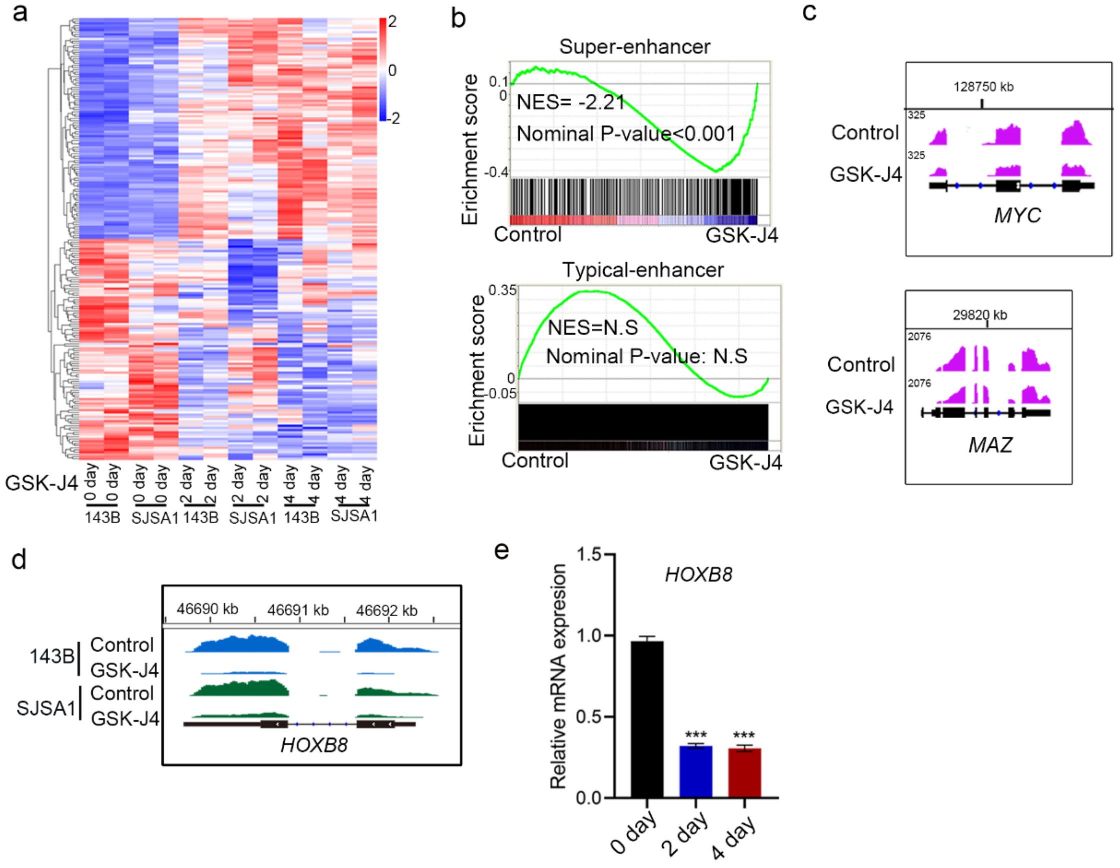

Figure S7. Effects of GSK-J4 on transcription of SE-driven genes in osteosarcoma cells. Related to Figure 5.

a) Heatmap showing shared top differential genes of 143B and SJSA1 cells upon 10 μM GSK-J4 treatment.

b) GSEA analysis plot of the downregulated SE-associated and TE-associated gene sets compiled from 143B and SJSA1 cells derived from GSK-J4 treatment (10 μM) versus that derived from control.

c) RNA-seq data showing the mRNA profiles of MYC or MAZ locus from control and 10 μM GSK-J4-treated 143B cells.

d) RNA-seq data showing the mRNA profiles of HOXB8 locus from control and 10 μM GSK-J4-treated 143B and SJSA1 cells.

e) qPCR assay showing the expression of HOXB8 upon 10 μM GSK-J4 treatment in 143B cells.

*, P < 0.05; **, P < 0.01; ***, P < 0.001 is based on the Student's t test. All results are from more than three independent experiments. Values are mean ± SD.

Supplemental Figure 8

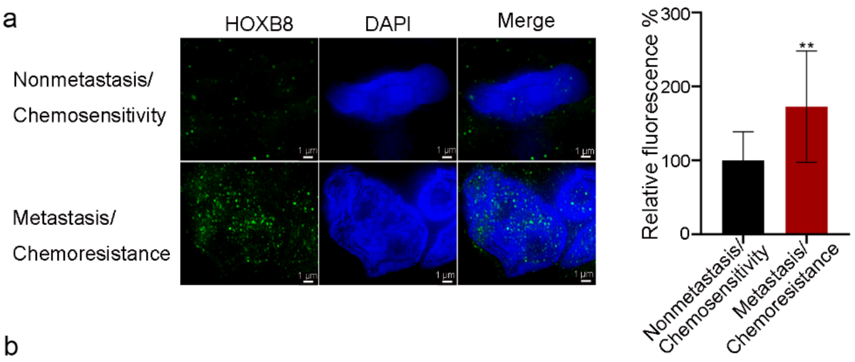

b

Variable	Univariate analysis			Multivariate analysis		
	Odds ratio	95% CI	p Value	Odds ratio	95% CI	p Value
Age						
≤18		1 (reference)				
>18	0.574	0.300-1.100	0.094		—	
Gender						
Female		1 (reference)				
Male	1.065	0.538-2.110	0.856		—	
Ennecking stage						
II		1 (reference)				
III	5.131	2.549-10.329	< 0.0001		—	
Lung metastasis						
Not present		1 (reference)				
Yes present	7.000	3.379-14.500	< 0.0001	6.440	2.223-18.653	0.001
Relapse						
Not present		1 (reference)				
Yes present	8.373	1.818-38.554	0.006	12.289	2.069-72.973	0.006
Chemoresistance						
Not present		1 (reference)				
Yes present	24.333	8.740-67.751	<0.0001	22.008	6.464-74.929	< 0.0001

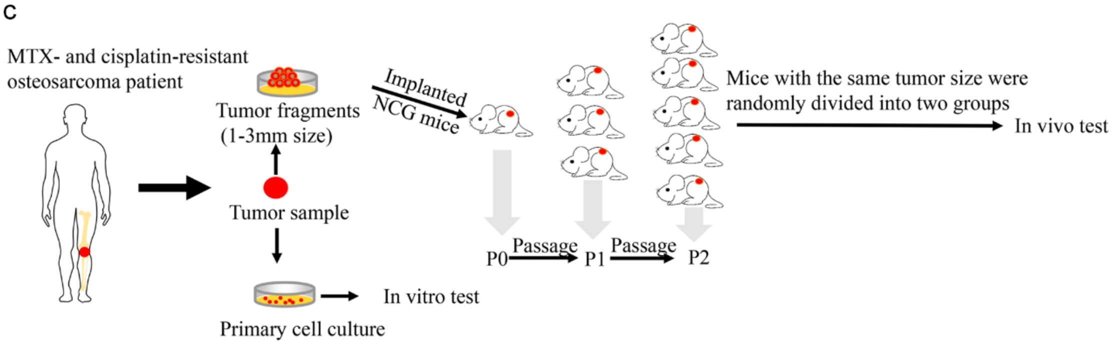

Figure S8. Pharmacological inhibition of CRC condensates confers additional advantages to circumvent chemo-resistance. Related to Figure 6.

a) Representative high-resolution images of HOXB8 condensates in non-metastatic osteosarcoma clinical specimens (n=3) and metastatic clinical specimens (n=3). Scale bar = 1 μm.

b) Univariate and multivariate analysis of clinical prognosis in 150 osteosarcoma patients.

c) Schematic flowchart showing the generation and application of PDX models in osteosarcoma.

*, $P < 0.05$; **, $P < 0.01$; ***, $P < 0.001$ is based on the Student's t test. All results are from more than three independent experiments. Values are mean ± SD.

Table S1. Patients' information.

Patients' information in Figure 1.

Sample	OS1	OS2	OS3	OS4
Gender	Male	Male	Male	Male
Operative age (Year)	13	15	10	21
Number of chemotherapy-before operation	4	4	5	3
Number of chemotherapy-after operation	13	14	15	6
Pathological diagnosis	OS	OS	OS	OS
Primary site	tibia	femur	femur	femur
Tumor metastatic sites	Pulmonary	Pulmonary	Pulmonary	Pulmonary
Recurrent site		femur		

Patients' information in Figure 7.

Sample	Age (year)	Gender	Stage	Metastasis	Relapse	Chemoresistance	IHC score
1	26	Female	2	No	No	No	2
2	29	Female	2	No	No	No	2
3	14	Male	2	No	No	No	6
4	20	Male	3	Yes	No	No	0
5	60	Male	3	Yes	No	No	0
6	23	Male	3	Yes	Yes	Yes	6
7	22	Male	2	No	No	No	2
8	16	Female	2	No	No	No	1
9	14	Male	2	No	No	No	0
10	10	Female	2	Yes	No	No	3
11	21	Female	2	Yes	No	Yes	9
12	18	Female	2	No	No	No	9
13	6	Male	2	Yes	No	Yes	4
14	12	Male	2	No	No	No	0
15	49	Male	3	Yes	Yes	Yes	9
16	17	Female	2	No	No	No	1
17	30	Male	2	No	No	No	0
18	11	Male	3	Yes	No	Yes	9
19	24	Male	2	Yes	No	Yes	4
20	24	Male	2	No	No	No	1
21	15	Male	2	No	No	No	0
22	15	Male	2	No	No	No	4
23	9	Male	2	No	No	No	0
24	14	Male	2	Yes	Yes	No	1
25	8	Male	3	Yes	No	Yes	6
26	19	Male	2	No	No	No	0

Sample	Age (year)	Gender	Stage	Metastasis	Relapse	Chemoresistance	IHC score
27	29	Female	2	No	No	No	0
28	35	Male	2	Yes	No	No	1
29	26	Male	2	No	No	No	0
30	64	Female	3	Yes	No	Yes	9
31	29	Female	2	Yes	No	No	0
32	20	Male	2	No	No	No	1
33	11	Male	2	Yes	No	Yes	9
34	14	Female	2	No	No	No	1
35	23	Female	2	Yes	Yes	Yes	9
36	14	Female	2	Yes	No	No	3
37	38	Female	2	No	No	No	1
38	11	Female	2	Yes	No	No	0
39	11	Female	2	Yes	No	No	1
40	17	Male	3	Yes	No	No	6
41	15	Female	2	No	No	Yes	0
42	17	Male	2	Yes	Yes	No	2
43	12	Female	2	No	No	No	1
44	23	Female	3	Yes	No	Yes	9
45	9	Male	3	Yes	Yes	No	4
46	16	Male	2	Yes	No	Yes	4
47	19	Male	3	Yes	No	No	4
48	19	Male	2	No	No	Yes	0
49	14	Male	2	No	No	No	0
50	16	Female	3	Yes	No	Yes	2
51	11	Female	3	Yes	No	Yes	6
52	21	Male	3	Yes	Yes	Yes	6
53	15	Female	3	Yes	Yes	Yes	9
54	17	Male	2	No	No	No	0
55	17	Male	2	No	No	No	2
56	7	Male	2	No	No	Yes	3
57	16	Male	2	No	No	No	1
58	15	Female	2	Yes	No	Yes	3
59	14	Female	2	No	No	No	0
60	21	Male	2	No	No	No	1
61	19	Male	2	Yes	No	Yes	6
62	13	Female	3	Yes	No	No	6
63	25	Female	3	Yes	No	No	0
64	51	Male	3	Yes	No	Yes	9
65	11	Male	2	Yes	No	No	4
66	9	Male	2	Yes	No	No	0
67	35	Male	3	Yes	No	Yes	0
68	50	Male	2	No	Yes	Yes	6
69	15	Male	2	Yes	Yes	Yes	6

Sample	Age (year)	Gender	Stage	Metastasis	Relapse	Chemoresistance	IHC score
70	18	Male	2	Yes	No	No	9
71	24	Male	2	No	No	No	1
72	14	Male	3	Yes	Yes	No	1
73	22	Female	2	No	Yes	Yes	4
74	16	Male	3	Yes	Yes	Yes	9
75	24	Male	2	No	No	No	2
76	17	Male	3	Yes	No	No	4
77	18	Male	3	Yes	No	No	4
78	21	Male	3	Yes	Yes	No	2
79	12	Male	2	No	No	No	2
80	10	Male	2	No	No	No	2
81	20	Male	3	Yes	Yes	Yes	3
82	12	Male	3	Yes	No	No	2
83	12	Male	3	Yes	No	No	2
84	22	Male	3	Yes	No	No	1
85	23	Female	2	No	No	No	2
86	10	Male	2	No	No	No	1
87	13	Male	3	Yes	No	Yes	6
88	21	Male	2	No	No	No	3
89	31	Male	2	No	No	No	2
90	21	Female	2	No	No	No	2
91	20	Female	3	Yes	Yes	Yes	2
92	14	Male	2	No	No	No	3
93	17	Male	3	Yes	No	No	1
94	28	Female	2	No	No	No	1
95	9	Male	3	Yes	No	No	2
96	29	Male	3	Yes	Yes	Yes	9
97	22	Female	2	No	Yes	Yes	3
98	19	Male	3	Yes	No	No	1
99	30	Male	2	No	No	No	1
100	19	Male	3	Yes	No	No	1
101	23	Male	2	No	Yes	Yes	6
102	12	Female	3	Yes	No	No	3
103	16	Female	3	Yes	No	Yes	6
104	29	Male	2	No	No	No	2
105	29	Female	3	Yes	Yes	No	2
106	6	Male	2	No	No	No	1
107	67	Male	3	Yes	No	Yes	6
108	16	Male	2	No	No	No	3
109	34	Female	2	No	No	No	2
110	18	Male	3	Yes	Yes	Yes	6
111	12	Female	2	No	Yes	No	2
112	19	Male	2	No	No	No	1

Sample	Age (year)	Gender	Stage	Metastasis	Relapse	Chemoresistance	IHC score
113	15	Male	2	No	No	No	2
114	35	Male	3	Yes	No	Yes	4
115	52	Female	2	No	No	No	2
116	17	Male	2	No	No	No	1
117	14	Female	2	No	No	No	3
118	45	Male	2	No	No	No	2
119	28	Female	2	No	Yes	No	1
120	51	Female	2	No	No	No	1
121	20	Male	2	No	No	No	2
122	18	Male	3	Yes	No	Yes	6
123	14	Male	2	No	No	No	3
124	12	Male	3	Yes	Yes	Yes	6
125	27	Male	2	No	No	No	3
126	18	Male	3	Yes	No	No	6
127	11	Male	2	No	No	No	2
128	14	Male	2	No	No	No	3
129	16	Female	3	Yes	Yes	Yes	6
130	12	Male	3	Yes	No	Yes	6
131	22	Male	2	No	No	No	2
132	38	Male	3	Yes	No	Yes	6
133	32	Female	3	Yes	No	No	4
134	15	Female	2	No	No	No	3
135	15	Male	3	Yes	No	Yes	4
136	47	Male	2	No	No	No	2
137	14	Male	3	Yes	Yes	Yes	4
138	19	Male	3	Yes	No	No	6
139	16	Male	3	Yes	No	No	2
140	13	Male	3	Yes	No	Yes	9
141	18	Female	3	Yes	No	Yes	9
142	19	Male	3	Yes	No	No	3
143	10	Female	3	Yes	No	No	2
144	16	Male	3	Yes	No	No	2
145	12	Female	3	Yes	No	Yes	4
146	17	Male	2	No	No	No	2
147	26	Male	3	Yes	No	No	4
148	22	Female	3	Yes	No	Yes	6
149	18	Female	3	Yes	Yes	Yes	3
150	16	Male	3	Yes	Yes	Yes	6

Table S2. shRNAs sequences and Primers used in this study

Genes	shRNA sequences
HOXB8 shRNA-1	5' AGTACGCAGACTGCAAGCTTG 3'
HOXB8 shRNA-2	5' GAGTTCCTATTTAATCCCTAT 3'
**Genes**	**Real-time PCR primers**
human HOXB8-forward	5' GTCCCTGCGCCCCAATTATTA 3'
human HOXB8-reverse	5' GCCCGTGGTAGAACTCCTG 3'
human MYC-reverse	5' TTGGACGGACAGGATGTATGC 3'
human MYC-forward	5' GTCAAGAGGCGAACACACAAC 3'
human MAZ-reverse	5' AAGCTGCCTCACATTTCTCAC 3'
human MAZ-forward	5' ACCACCTGAACCGACACAAG 3'
human FOSL1-forward	5' CACTCCAAGCGGAGACAGAC 3'
human FOSL1-reverse	5' AGGTCATCAGGGATCTTGCAG 3'

Table S3. Antibodies and compounds used in this study

Regent or Resource	Source	Identifier
**Antibody**		
Rabbit polyclonal anti-H3K27Ac	Abcam	Cat# ab4729
Rabbit polyclonal anti-H3K4me3	Abcam	Cat# ab8580
Rabbit polyconal anti-normal rabbit IgG	Cell Signaling technology	Cat# 2729
Rabbit monoclonal anti-Flag	Cell Signaling technology	Cat# 4793
Mouse monoclonal anti-Beta-catenin	HuaAn Biotechnology	Cat# M1405-6
HRP-conjugated Affinipure Goat anti-Rabbit IgG	Proteintech	Cat# SA00001-2
Rabbit polyclonal anti-Ki67	HuaAn Biotechnology	Cat# ER1802-31
Rabbit polyclonal anti-HOXB8(ChIP)	Abcam	Cat# ab125727
Rabbit polyclonal anti-HOXB8(IHC)	Bioss	Cat# bs-6539R
Rabbit Polyclonal anti-HOXB8(IP)	LSBio	Cat# LS-C487001
Rabbit Polyclonal anti-FOSL1(IF)	Abcam	Cat# ab124722
Rabbit polyclonal Anti-HA tag(HRP)	Abcam	Cat# ab128131
Mouse monoclonal anti-Beta actin	Proteintech	Cat# 60008
Mouse monoclonal anti-RNA polymerase II	Millipore	Cat# 05-623B
**Chemicals, Peptides, and Recombinant Proteins**		
Cisplatin	Selleck	Cat# 15663-27-1
Methotrexate	Selleck	Cat# S1210
B27	Invitrogen	Cat# 17504-044
Human aFGF	Beyotime	Cat# P5448
Human bFGF	PeproTech	Cat# AF-100-18B-100
Human EGF	PeproTech	Cat# AF-100-15-500
GSK-J4	Selleck	Cat# S7070

Supplementary Materials and Methods

Quantitative RT-PCR

Total RNA of cells was extracted using TRIzol (Invitrogen, 15596018), and 1 μg RNA was reverse-transcribed into cDNA using HiScript II Q RT SuperMix for qPCR (Vazyme, R223-01). For quantitative PCR, SYBR Green Ⅰ RT-PCR kit (GENSTAR, A314) was used as per manufacturer's instructions. For detection of the expression levels of target genes, GAPDH was used as internal control, and the ΔCt values were calculated for analysis. Sequences of other primers are shown in **Supplementary Table S2**.

Western Blot

Cells were lysed in RIPA buffer with protease inhibitor cocktail (Roche 4693132001). Samples were diluted with 0.25 volume to 5× SDS-PAGE Sample Buffer (GenStar). Gel electrophoresis was performed using SDS-PAGE, and proteins were transferred to Immun-Blot PVDF Membrane (Bio-Rad). Membranes were incubated with following primary antibodies **(Supplementary Table S3)** for 16–20 h at 4°C.

Immunocytochemistry

After fixation with 4% paraformaldehyde, osteosarcoma tissue was prepared into paraffin sections. Paraffin sections were dewaxed, hydrated, antigen repaired, and followed by blocking in 10% BSA for 40 min at 37 °C. Tissue samples were incubated with HOXB8 antibody (C36B11, Cell Signaling, 1:200) or Ki67 antibody (ab154985, Abcam, 1:400) in PBS overnight at 4°C. The samples incubated overnight were followed by incubation for 40 min at 37°C with horseradish marker sheep immunoglobulin polymer. The incubated samples were rinsed three times with PBS and added to DAB developer for 5–20 min at room temperature. The samples were re-dyed for 40 s with hematoxylin solution after the water was used to stop the DAB dyeing. The stained samples

were dehydrated, sealed, and observed under a microscope.

The scoring system is two pathologists independently and blind observed at least 5 fields of the sections under a 20-fold microscope, and scored the immunohistochemical staining intensity and positive cells of tumor cells. Staining intensity score: 0 points for no staining, 1 point for light yellow, 2 points for tawny and 3 points for brown; Positive cell area percentage score: 0 points for no positive tumor cells, 1 point for < 30% positive tumor cells, 2 points for 30%~60% positive tumor cells, and 3 points for more than 60% positive cells. The product of the score values was used as the score of immunostaining score, with a total of 7 score level of 0, 1, 2, 3, 4, 6 and 9. The patients were divided into two groups with high expression (immunostaining score greater than or equal to 4) and low expression (immunostaining score less than 4) through the median patient score, and then statistically analyzed.

Immunofluorescence

Osteosarcoma tissues from patients or mice were fixed with 4% paraformaldehyde, dehydrated with 30% sucrose, embedded with OCT and then cut using a cryostat (Thermo NX50). 143B cells grown on confocal dish (NEST, 801002). After three washes in TBS for 5min, cells were fixed with 4% paraformaldehyde. Frozen sections of osteosarcoma tissues and 143B cells were immunostained as follows. Following three washes in TBS for 5 min, samples were permeabilized with 0.3% triton X100 (Sigma Aldrich, X100) in TBS at RT for 30 min and then blocked with goat serum at RT for 1 h. Samples were incubated with HOXB8 antibody (bs-6539R, Bioss Antibody, 1:100) or FOSL1 antibody (ab124722, Abcam, 1:1000) in 0.2% BSA and 0.1% Triton X-100 in TBS (pH 7.4) overnight at 4°C. After three washes in TBS with 0.05% Tween 20, the sections were incubated with Alexa 488-conjugated secondary antibodies diluted in the same buffer as the primary antibodies for 1 h at RT. All sections were counterstained with DAPI. Following three additional

washes, the sections were coverslipped with mounting media and visualized with a super-resolution microscopy (A1R N-SIM N-STORM, Nikon, Japan). Immunofluorescence quantification was performed using ImageJ software.

Gene ontology analysis

Gene lists were imported into gProfiler to generate enrichment scores for all GO, Kyoto encyclopedia of genes and genomes (KEGG), and REACTOME gene sets according to the recommended settings for gProfiler (http://baderlab.org/ Software/EnrichmentMap/GProfilerTutorial/). Cytoscape (v3.2.1) and the Enrichment Map55 plug-in were used to generate networks for enriched gene sets with a false discovery rate cutoff of < 0.05.

Cell viability assay

The proliferation rate of osteosarcoma cells was determined using CellTiter-Glo® (CTG) Luminescent Cell Viability Assay (Promega, USA) following the user’s manual. In brief, cells were seeded in 96-well plates at a density of 2×10^3 cells/well and were incubated at 37°C in a humidified 5% CO_2 atmosphere. At 0, 12, 24, 36, 48, 60, and 72 h, the culture medium was discarded and 50 µL of CTG solution was added into each well, followed by incubation for 30 min on an orbital shaker at room temperature to induce cell lysis. The staining intensity in the medium was detected by measuring the absorbance at 450 nm for the optical density.

Transwell migration assays

Osteosarcoma cells (2,000–3,000) were re-suspended in DMEM without FBS, and the cell suspensions were uniformly added to the upper compartment covered by Matrigel. At the same time, 800 µl DMEM containing 10% FBS was added to the 24-well culture plate. The culture medium was discarded after 24 h, and the polycarbonate membrane chamber adsorbed with cells was fixed

with methanol for 10 min follow by staining with 0.5% crystal violet for 1 h. The polycarbonate membrane chamber was cleaned with water three times, the excess crystal violet was wiped off with a cotton ball, and the membrane was observed under the microscope.

RNA-seq data analysis

Trim_galore and Fastqc were used for trimming adapters and filtering raw sequencing reads. Sequencing reads were aligned to the UCSC hg19 human genome reference using hisat2. All unmapped reads, non-uniquely mapped reads and PCR duplicates were removed. High quality and uniquely aligned reads were counted at gene regions using the package Gfold based on Gencode Human GRCh37 annotations. Differential gene expression analysis between groups was performed using the R/Bioconductor package DESeq with contrast adjustment for multiple groups comparison. GO and KEGG pathway enrichment analysis were done using R/Bioconductor package clusterProfiler. Heatmaps were drawn using the R package Pheatmap and Ggplot2.

ATAC-seq chromatin preparation

Nuclei were prepared from 5×10^4 cells by spinning at 600 × *g* for 10 min at 4°C, followed by a PBS wash and centrifugation at 600 × *g* for 5 min. Cells were lysed using ice-cold lysis buffer (10 mM Tris-HCl, pH 7.4, 10 mM NaCl, and 3 mM $MgCl_2$, 0.1%), and centrifuged for 10 min at 600 × *g* at 4°C. The supernatant was removed, and the pellet was re-suspended in 50 μL transposase mix (25 μL 2× TD buffer, 2.5 μL transposase, and 22.5 μL water) (FC-121-1030 Illumina) for 30 min at 37°C. Library amplification was performed using the NEBnext High Fidelity 2× PCR Master Mix (#M0541S, New England Biolabs) according to previously published PCR conditions. PCR reactions were purified using a QIAGEN MiniElute kit and a following size selection step using standard gel extraction protocol to isolate a ~240–360 bp DNA band. ATAC-seq library preparations were sequenced using single-end 50-bp reads on the Illumina HiSeq 2000 platform. Raw reads were

adaptor-trimmed using Trim Galore (v0.2.5) and aligned to the genome with Bowtie (v1.0.1) with the m1 option enabled to allow only uniquely aligned high-quality reads. Peaks were called using the MACS2 software (v2.1.0.20140616) with the options −q 0.05 to retain significant peaks and shiftsize 50 to account for the transposase fingerprint, while default parameters were used for other options.

Protein binding study using Bio-Layer Interferometry

The binding of GSK-J4 to the HOXB8-IDR, HOXB8-full length and FOSL1-full length was carried out using Bio-Layer Interferometry instrument Octet Red96e with Streptavidin sensors. At first, proteins and biotin reacted at room temperature for 30min in a ratio of 1:1. After that, the free biotin was removed by desalting gravity column. Sensors were equilibrated offline in PBS for 10 min and then monitored on-line for 60 s for baseline establishment. For binding measurement, sensors were loaded with the biotinylated HOXB8-IDR, HOXB8-full length or FOSL1-full length, afterward they were transferred to PBS for 60 s for baseline establishment and then to ligand solutions for association for 60 s. Afterward sensors were transferred to PBS for 60 s for off-rate measurement. Kinetics data were fit using a 1:1 binding global model of data analysis software provided by ForteBio.
